# *Berberis microphylla* G. Forst (Calafate) Berry Extract Reduces Oxidative Stress and Lipid Peroxidation of Human LDL

**DOI:** 10.3390/antiox9121171

**Published:** 2020-11-24

**Authors:** Lia Olivares-Caro, Claudia Radojkovic, Si Yen Chau, Daniela Nova, Luis Bustamante, Jose Yamil Neira, Andy J. Perez, Claudia Mardones

**Affiliations:** 1 Departamento de Análisis Instrumental, Facultad de Farmacia, Universidad de Concepción, Concepción 4070386, Chile; liaolivares@udec.cl (L.O.-C.); daninovabaza@gmail.com (D.N.); lbustamante@udec.cl (L.B.); yneira@udec.cl (J.Y.N.); aperezd@udec.cl (A.J.P.); 2 Departamento de Bioquímica Clínica e Inmunología, Facultad de Farmacia, Universidad de Concepción, Concepción 4070386, Chile; cradojkovic@udec.cl (C.R.); schau@udec.cl (S.Y.C.)

**Keywords:** *Berberis microphylla* G. forst, Ultra Liquid Chromatography with diode array detector coupled to Quadrupole-Time of Fly Mass Spectrometry (UHPLC-DAD-QTOF), phenolic compounds, human umbilical vein endothelial cells (HUVEC), cardiovascular disease, antioxidant capacity

## Abstract

Calafate (*Berberis microphylla* G. Forst) is a Patagonian barberry very rich in phenolic compounds. Our aim was to demonstrate, through in vitro models, that a comprehensive characterized calafate extract has a protective role against oxidative processes associated to cardiovascular disease development. Fifty-three phenolic compounds (17 of them not previously reported in calafate), were tentatively identified by Ultra-Liquid Chromatography with Diode Array Detector, coupled to Quadrupole-Time of Fly Mass Spectrometry (UHPLC-DAD-QTOF). Fatty acids profile and metals content were studied for the first time, by Gas Chromatography Mass Spectrometry (GC-MS) and Total X-ray Fluorescence (TXRF), respectively. Linolenic and linoleic acid, and Cu, Zn, and Mn were the main relevant compounds from these groups. The bioactivity of calafate extract associated to the cardiovascular protection was evaluated using Human Umbilical Vein Endothelial Cells (HUVECs) and human low density lipoproteins (LDL) to measure oxidative stress and lipid peroxidation. The results showed that calafate extract reduced intracellular Reactive Oxygen Species (ROS) production (51%) and completely inhibited LDL oxidation and malondialdehyde (MDA) formation. These findings demonstrated the potential of the relevant mix of compounds found in calafate extract on lipoperoxidation and suggest a promising protective effect for reducing the incidence of cardiovascular disease.

## 1. Introduction

Polyphenols are natural antioxidants that reduce oxidation processes and inhibit the production of free radicals [1]. They are commonly found in vegetables, fruits, and many other foods; moreover, they are responsible for the color and flavor of these foods [2]. Phenolic compounds have been described as molecules with antibiotic, antitumoral, antioxidant, and antiviral properties, among other bioactivities [3]. Many studies have demonstrated that certain polyphenols (delphinidin, resveratrol, quercetin, etc.) can contribute to the prevention of chronic non-communicable diseases, such as obesity, Alzheimer’s disease, cancer, and cardiovascular disease (CVD) [4].

CVD is the main cause of death both in Chile and around the world [4]: 27% of total deaths in Chile (Ministerio de Salud de Chile, MINSAL) and 31% worldwide (World Health Organization, WHO) are caused by stroke or acute myocardial infarction. Hypertension, dyslipidemias, obesity, and metabolic syndrome, among others, have been described as risk factors of CVD, and their common denominator is endothelial dysfunction [1]. This condition is produced by an imbalance in the normal function of endothelial cells, which leads to the hallmark of CVD, a vasoconstricted, inflammatory, and pro-coagulative state [5]. The chronic exposure to harmful physical and chemical stimuli, such as oxidized low density lipoproteins (LDL), can result in endothelial dysfunction by increasing levels of reactive oxygen species (ROS) and oxidative stress in endothelial cells [5].

According to several studies, the consumption of polyphenolic compounds can reduce CVD risk [6]. The PREDIMED (Prevención con Dieta Mediterránea) study demonstrated an inverse relationship between anthocyanin consumption and CVD risk [6]. Notably, decreased ROS production, lower expression of inflammatory markers, and decreased blood pressure have been described as important effects of certain polyphenols [4,7].

Calafate is an endemic evergreen shrub of the Berberidaceae family from Chilean-Argentine Patagonia. The shrub is 1 to 2 m high, with leaves grouped in rosettes and yellowish spines. Its flowers are solitary, with a long peduncle that is born from each rosette of leaves. Its bloom is in September and the fruit is edible. The shrub produces a dark-skinned barberry with a high polyphenol content and antioxidant capacity [8,9,10], which are normally consumed fresh or as juices, marmalade, and infusions. Anthocyanins, hydroxycinnamic acids, and flavonols are the main phenolic compounds described in the fruit [11,12,13]. Anthocyanins can trap free radicals, reducing oxidative stress, a common factor in the development of chronic non-communicable diseases [14]. In a study carried out by Ramirez et al. [14], using erythrocytes obtained from healthy humans, researchers demonstrated that a methanolic calafate extract inhibited 90% of the lipidic peroxidation caused by tert-butylhydroperoxide. Lipidic peroxidation is crucial in the induction and/or propagation of oxidative stress-related diseases [15]. Additionally, calafate berry extract increased the ratio of reduced/oxidized glutathione (GSH/GSSG), which led to decreased oxidative stress in murine adipocytes treated with macrophage-conditioned medium [16]. Another study reported inhibition of tumor necrosis factor *alpha* (TNF-α) gene expression (a cytokine related to proinflammatory state) in murine macrophages stimulated with lipopolysaccharides in the presence of calafate extract [17]. Finally, in a recent study, Calfío and Huidobro-Toro described a vasodilatory effect of a hydroalcoholic calafate berry extract, which was 89.6% (*p* < 0.01) inhibited by N-Nitro-L-Arginina (L-NNA) (150 µM), an inhibitor of endothelial nitric oxide synthase (eNOS) [18]. These findings suggest that nitric oxide, one of the most important endothelium-derived vasodilatory molecules, participates in the vascular response induced by calafate. Altogether, these studies suggest that calafate can contribute to reduce endothelial dysfunction, which is the basis of cardiovascular disease. However, none of them included the study of lipid peroxidation based on a LDL model, which is directly associated with the atheroma formation [1]. 

Some fatty acids, mainly poly-unsaturated, are other compounds associated with the prevention of CVD through inhibitory effects on inflammation, oxidation, and thrombosis [19]. On the other hand, certain metals (Mn, Cu, and Zn) have shown an activating effect on enzymes involved in antioxidant protection in humans [20]. To our knowledge, neither fatty acids nor metals have been studied in calafate. 

To fill this gap, we studied the effect of a comprehensive characterized calafate extract against oxidative stress using Human Umbilical Vein Endothelial Cells (HUVECs) and lipoperoxidation on human LDL, as biological models directly related with CVD. The main objective was to demonstrate that the relevant mix of compounds found in calafate extract, determined by Ultra-Liquid Chromatography with Diode Array Detector, coupled to Quadrupole-Time of Fly Mass Spectrometry (UHPLC-DAD-QTOF), Gas Chormatography Mass Spectrometry (GC-MS), and Total Reflection X-ray Fluorescence (TXRF), reduces ROS production and lipoperoxidation, both processes related with the prevention of cardiovascular disease incidence.

## 2. Materials and Methods 

### 2.1. Reagents and Vegetable Material

Reagents: Commercially available delphinidin 3-glucoside, 3-cafeoylquinic acid, BF3-methanol 10% LiChropure for fatty acid derivatization, Miristic acid d27, and Supelco 37 Component fatty acid methyl esters (FAME) Mix were obtained from Sigma-Aldrich (Steinheim, Germany). Formic acid, ammonia (25%), sodium hydroxide, sodium chloride, hydrochloric acid, acetonitrile, methanol, ethanol, water (LC-MS grade or hypergrade), and Hexane (SupraSolv^®^, city, state, country) for gas chromatography, Dimethyl sulfoxide (DMSO), and Vitamin C were provided by Merck (Darmstadt, Germany). Chloroform (Optima^®^ GC/MS) and BCA (bicinchoninic acid) Protein Assay Kit were provided by Fisher Scientific (Waltham, MA, USA). Solid phase extraction (SPE) 150 mg Oasis MCX 6 cm^3^ mixed phase cartridges were from Waters (Milford, MA, USA). 2′,7′-Dichlorodihydrofluorescein diacetate (H_2_DCF-DA) was obtained from Invitrogen (Waltham, MA, USA) and malondialdehyde (MDA) was purchased from Sigma-Aldrich (St. Louis, MO, USA). M199 medium, fetal bovine serum (FBS), and other cell culture reagents were obtained from Gibco Life Technologies (Grand Island, NY, USA). Penicillin-streptomycin solution and Trypsin 0.25% were purchased from HyClone Co. (Logan, UT, USA). 3-(4,5 Dimethylthiazol 2)-2,5-diphenylterazolium bromide (MTT) was purchased from Cayman Chemical Company (Ann Arbor, MI, USA).

Vegetable material: Calafate berries (*Berberys microphylla* G. Forst) were collected near Punta Arenas, Chile (53.1548309–70.911293). Berries were collected between February and May 2019 at maturity stage and were frozen at –80 °C until their analyses. Approximately 1 Kg of fruit was collected, which was homogenized before analysis. A taxonomical identification, by comparison of the species with those from the University of Concepcion Herbarium, was carried out in order to assure the identity of the samples. The moisture (%) of the fruits was 69.4% ± 2.7%.

### 2.2. Instrumentation 

An ultrasonic bar homogenizer Cole Palmer series 4710 (Chicago, IL, USA), a mechanical shaker (Edyman KL2), a desktop centrifuge Heraeus-Christ GmbH (Osterode, Germany), an Alpha 2–4 LD plus lyophilizing system from Martin Christ (Osterode, Germany), a centrifuge Heraeus-Fresco17 from Thermo Fisher (Waltham, MA, USA), a laboratory stirrer/hotplate CORNING model PC420 (Durham, NC, USA), an analytical balance from Denver Instrument Company (NY, USA), a rotavapor with a V-700 vacuum pump and V-850 controller system from Büchi (Flawil, Switzerland), and a MM 400 mixer mill from Retsch GmbH (Haan, Germany) were used for sample preparation. An Epoch™ microplate reader from Biotek Instruments (Winooski, VT, USA) was used for antioxidant and cellular assays. A Beckman Optima ^TM^ LE 80 K (Palo Alto, CA, USA) was used for ultracentrifugation.

The identity assignment of phenolic compounds was carried out with a UHPLC-DAD Bruker Elute LC system, coupled in tandem with a Q-TOF spectrometer Compact, Bruker (Bremen, Germany). The control system used was Compass HyStar (Bruker), the acquisition software was Bruker otofControl 4.1.402.322-7977-vc110 6.3.3.11, and data analysis was performed with Compass DataAnalysis 4.4 software (Bruker Daltonik GmbH, Germany). 

The quantitative analysis of phenolic compounds was carried out with a Shimadzu HPLC system equipped with a quaternary LC-10ADVP pump, an FCV-10ALVP elution unit, a DGU-14A degasser unit, a CTO-10AVP oven, and a ultraviolet-visible diode array spectrophotometer model SPD-M10AVP. The control system and data collection were performed using Shimadzu Chromatography Data System CLASS-VP software (Kyoto, Japan).

The fatty acid profile was determined using an Agilent 7890A GC (Palo Alto, CA, USA) with a multimode injector, interfaced to an Agilent 7000 GC/MS Triple Quad detector, fitted with an Agilent CTC PAL autosampler and controlled by Agilent MassHunter GC/MS acquisition software (Version B.05.00/Build 5.0.291.0). 

Metal profile analysis was performed using a benchtop TXRF system (S4 TStar, Bruker^®^ AXS Microanalysis GmbH, Berlin, Germany) equipped with a 50 W X-ray tube with a molybdenum (Mo) anode and a multilayer monochromator (17.5 keV).

### 2.3. Comprehensive Chemical Characterization of Calafate

#### 2.3.1. Extraction Protocols 

The extraction was carried out according to a protocol previously described by Ruiz et al. [11]. Briefly, 5 g of calafate berries were triturated, mixed with 10 mL of methanol/formic acid (97:3 ratio), and sonicated (60 s, 40% A). The sample was then stirred for 16 h and centrifuged at 1730× *g* for 10 min. Subsequently, the supernatant was removed and added to a 25 mL flask. An additional 5 mL of solvent was added to the sample and stirred for 30 min; the sample was then centrifuged, and the supernatant was transferred to a 25 mL flask. This procedure was repeated 3 times. Finally, the flask was filled with the same solvent, obtaining the crude extract. Additionally, the same procedure was developed using ethanol/formic acid (97:3 ratio), in order to compare the effect of the extraction solvents (methanol vs. ethanol) on the polyphenolic composition and on the biological assays (Raw extracts).

Hydroxycinnamic acid determination required a purification step using solid phase extraction with cation exchange columns (Oasis MCX Water, USA), as previously described by Ruiz et al. (Purified extract) [12]. Briefly, 1 mL of crude extract was evaporated and resuspended in 5 mL of 0.1 M HCl. Subsequently, the MCX SPE-column was conditioned with 5 mL of methanol and 5 mL of ultrapure water. The resuspended crude extract was loaded onto the SPE column and subsequently washed with 5 mL of 0.1 M HCl and 5 mL of ultrapure water. The elution of hydroxycinnamic acids was done with 5 mL of solvent and repeated 3 times, obtaining an extract rich in hydroxycinnamic acids (Purified extract).

Raw extracts (methanolic and ethanolic) were used for the analysis of anthocyanins in fruit by LC-MS, metal profiling by TXRF, fatty acids by GC-MS, and in vitro assays. Purified extract was used for hydroxycinnamic acid identification and quantitation.

#### 2.3.2. Identification and Quantitation of Anthocyanins

The identification of anthocyanins by LC-MS was based on the method previously described by Ruiz et al. [12]. This method uses a mobile phase with lower formic acid concentration than the common method for anthocyanin [8], because the ESI system of the spectrometer does not allow the use of high formic acid concentrations (Compact User Manual, Bruker Daltonik, GmbH). Chromatographic separation conditions were as follows: a Kromasil column 100 5 C18 250 × 4.6 mm with a C-18 precolumn (Nova-Pak Waters, 22 × 3.9 mm, 4 μm), mobile phase A: formic acid 0.1% in water, and mobile phase B: 0.1% formic acid in acetonitrile. A flow rate of 0.3 mL/min and an injection volume of 10 μL were used. The acetonitrile gradient ranged from 15% to 25% for 14 min, from 25% to 35% for 11 min, from 35% to 100% for 1 min, 100% for 9 min, and from 100% to 15% for 1 min with a stabilization period of 25 min. The spectrophotometric detection was set at 520 nm, and MS detection was set using the base peak chromatogram (BPC), both obtained in the LC-MS. The MS conditions for anthocyanin identification were as follows: positive ionization ESI +4500 V, dry gas: 9 L/min, nebulizer: 4 Bar, T : 200 °C, end capillary 500 V, collision energy at 10–25 eV in stepping mode, Auto MS/MS mode (4 precursor/cycle), 50–1500 *m*/*z* (scan 0.2 s centroid mode), and internal calibration using sodium formate (10% formic acid, 1 M) with a mass accuracy <3 ppm.

Quantitative analysis was carried out using a Shimadzu HPLC system by external calibration curve using a delphinidin 3-glucoside standard at 518 nm, as described by Ruiz et al. [8]. 

#### 2.3.3. Hydroxycinnamic Acid Derivatives (HCAD) Analysis by LC-MS

Identification was performed as previously described Ruiz et al. [12]. The same column, precolumn, and chromatographic separation used for anthocyanin analysis were used. The spectrophotometric detection was set at 320 nm, and MS detection was set using the BPC, both obtained in the LC-MS. The same spectrometric conditions used in Section 2.3.2 were used, with the exception of negative ionization ESI −3500 V. The quantitative analysis was carried out using a Shimadzu LC-DAD system by an external calibration curve prepared with 3-caffeoylquinic acid standard and measured at 326 nm.

#### 2.3.4. Metal Profile Analysis

The identification and quantitation of metals in calafate was carried out by a previous siliconization procedure for TXRF measurement. For this purpose, 10 µL of silicone solution in isopropanol was dropped onto the surface of quartz carriers and dried at 110 °C on a hot plate. Separately, 10 μL of methanolic and ethanolic extracts (dilution 1/100) containing 0.5 mg/L Ga as an internal standard were dropped onto the siliconized carriers. After drying the samples at 80 °C, they were placed into the TXRF equipment. The characteristic radiation emitted by each element was detected by a silicon drift detector with an area of 60 mm^2^ and an energy resolution <145 eV, working at 50 kV and 750 µA in the air. The measurement live time was 1200 s. Elements were identified automatically by referring to the software database. The quantitative analysis was carried out by a standard internal method using Ga [21]. 

#### 2.3.5. Analysis of Fatty Acid Profile

The fatty acid profile and concentration were carried out on lyophilized calafate fruit and in the methanolic and ethanolic extracts (described in Section 2.3.1). The lyophilized fruit was homogenized using a ball mill (30 cycles/s for 1.5 min). Subsequently, 100 mg of homogenized calafate (or 250 μL of extracts) mixed with 1 mL of methanol/chloroform 2/1% *v*/*v* solution were transferred to a 1.5 mL centrifuge tube and shaken (ball mill 30 cycles/s during 1.5 min), followed by a centrifugation step (9600× *g* for 2 min at 4 °C). A mix of 780 μL of the resulting supernatant, 260 μL of chloroform, and 468 μL of water was transferred to a 4 mL vial and vortexed. Finally, 400 μL of the organic phase was transferred to a new 4 mL glass vial and evaporated under a nitrogen stream for derivatization.

Fatty acid methyl ester (FAME) derivatization was achieved using the AOAC 969.33 protocol for fatty acids [22] with Myristic-d-27 acid (75 mg/L) as the internal standard. The GC-MS analysis was performed using a 30 m × 0.25 mm × 0.2 μm ionic liquid SLB-IL-111 column from Supelco. The injection volume was 1 μL, the injection temperature was 250 °C, the split mode was 10:1, and the gas carrier flow was 1.5 mL/min. The oven temperature program started at 40 °C for 4 min, with a 5 °C/min ramp up to 260 °C for a total analysis time of 48 min. The transference line was set at 275 °C, with an ionization source of 70 eV and 230 °C, with a Quadrupole temperature of 150 °C. Mass spectra acquisition was performed in scan mode from 20 to 450 *m*/*z*, with 140 ms scan time. Fatty acids were identified by comparing the retention times (RT) with a FAME mix, the mass spectra, and NIST05 library.

Extracted ions at *m*/*z* 74 and 87 were used for the saturated and monounsaturated fatty acids. The ions at *m*/*z* 79 and 81 were used for the di- and tri-unsaturated fatty acids. The areas corrected by the internal standard of the samples and the standard mix (Supelco pn: CRM47885, XA18647V) were compared by applying a response factor method, according to the AOAC 996.06. The concentration of the different fatty acids was expressed as mg/g fresh weigh (FW) or mg/mL extract.

### 2.4. In Vitro Assays

All in vitro assays were carried out using the characterized extracts obtained in Section 2.3.1. (methanolic and ethanolic extracts).

#### 2.4.1. Antioxidant Capacity and Total Phenolic Compounds

Trolox equivalent antioxidant capacity assay by cupric-reducing antioxidant capacity (TEAC_CUPRAC_) and Trolox equivalent antioxidant capacity assay with 2,2′-azino-bis(3-ethylbenzothiazoline-6-sulfonic acid (TEAC_ABTS_) were determined as previously described by Ruiz et al. [8] and Ruiz et al. [11]. Trolox equivalent antioxidant capacity assay by oxygen radical absorbance capacity (TEAC_ORAC_) was carried out according to Ou et al. [23]. Total phenolic compounds were quantified using the Folin–Ciocalteu method [8], and all methodologies were carried out in 96-well microplates. 

#### 2.4.2. Cell Culture

HUVECs (Lonza Group) were cultured on gelatin-coated plastic dishes with M199 medium supplemented with 20% fetal bovine serum (FBS) at 37 °C in 5% CO_2_ [24]. For experiments, cells were recovered with Trypsin/ ethylenediaminetetraacetic acid (EDTA) and seeded in 96-well plates. All experiments were performed between passages 3–8 and treatments were added at 90% confluence. 

##### Viability Assay

Cell viability was determined by the MTT method [25]. HUVECs were seeded at a density of 30,000 cells/well into 96-well plates (100 µL) and were cultured for 1 day in M199 medium supplemented with 20% FBS. Then, cells were pretreated for 48 h with different dilutions of calafate extracts in culture medium supplemented with 2% FBS or with phosphate buffered saline (PBS) 10 mM (145 mM NaCl, 7.5 mM Na_2_HPO_4_, 2.5 mM NaH_2_PO_4_, pH 7.2–7.4), which was used as a positive control to induce cell death by serum deprivation. After the incubation period, cells were incubated with the MTT solution at a final concentration of 0.5 mg/mL for 4 h at 37 °C. Next, 85 µL of medium was removed and DMSO (50 µL) was added to each well. Absorbance was measured at 540 nm with a microplate reader. Viability was expressed as a percentage as compared to nontreated cells cultured in M199 medium with 2% FBS (viability control).

##### ROS Measurement

The intracellular ROS measurement was based on ROS-mediated conversion of a nonfluorescent H_2_DCFH-DA probe into dichlorofluorescein (DCF), whose intensity of fluorescence reflected enhanced oxidative stress. For these experiments, HUVECs were seeded in 96-well plates at a density of 30,000 cells/well, and after 1 day, they were incubated with calafate dilutions for another 24 h. After washes with PBS 10 mM, cells were incubated with H_2_DCFH-DA (10 μM) in 10 mM PBS at 37 °C for 45 min. At the end of the incubation period, cells were washed and stimulated with H_2_O_2_ (200 µM), and DCF fluorescence was measured at 540 nm (emission) and 485 nm (excitation) after 45 min with a microplate fluorescence reader. Cells exposed to H_2_O_2_ (200 µM) but not to calafate extract were used as a positive control of oxidative stress. The results were expressed as fold change of fluorescence intensity compared to non-stimulated HUVECs (negative control: cells not exposed to either calafate or H_2_O_2_) [5]. As an antioxidant control, vitamin C (250 µg/mL) was added to cell cultures at the same time as H_2_O_2._

#### 2.4.3. LDL Isolation 

Low density lipoprotein (LDL) extraction was done as described by Havel, Eder, and Bragdon [26]. Peripheral blood was obtained from healthy donors (6) after 10 h of starvation. All participants signed a written informed consent form before sample collection. LDL samples were collected, dialyzed in PBS 10 mM pH 7.4, and quantified by the BCA assay kit for further studies. This protocol conforms to the Declaration of Helsinki, and it was revised and approved by the University of Concepción Ethics Committee (CEBB-427-2-2019).

##### LDL Oxidation

LDL (500 µg/mL) was incubated at 37 °C in PBS 10 mM with or without CuSO_4_ (7 µM) (positive and negative controls, respectively), in a final reaction volume of 100 µL. For some experiments, different dilutions of calafate extract were incubated together with LDL and CuSO_4_. Kinetics of oxidation were recorded every minute by absorbance at 232 nm in a microplate reader [5].

##### MDA Assay 

To evaluate lipoperoxidation, LDL was incubated at 37 °C with CuSO_4_ (7 µM) (positive control) with or without calafate extract or an antioxidant control (vitamin C 250 µg/mL) for 1 h 20 min. Then, reaction was stopped with 1 µL of BHT 5% p/v. MDA quantification was carried out by the thiobarbituric acid reactive substances (TBARS) method as described by Richard in 1992, with some modifications [27]. Briefly, 100 µL of MDA standard or LDL, 100 µL trichloroacetic acid 10% *w*/*v*, and 800 µL thiobarbituric acid 0.53% *w*/*v* were added in cryotubes and vortexed. The cryotubes were boiled for 60 min at 95 °C, and the reaction was stopped in ice for 15 min. Finally, the mix was transferred to a 96-well plate and the absorbance was read at 540 nm. Quantification was carried out by external calibration with MDA standard. Negative control consisted in LDL incubated only with PBS 10 mM.

### 2.5. Statistical Analysis 

All results were expressed as arithmetic means and standard errors. Data were subjected to one-way analysis of variance (ANOVA) to evaluate the statistical significance of intergroup differences with Bonferroni post hoc tests, considering α < 0.05. Graphics were performed using GraphPad Prism software version 5.0 and statistical analyses were carried out using IBM SPSS software version 20.

## 3. Results and Discussion

### 3.1. Phenolic Profile in Calafate Fruit by UHPLC-DAD-ESI-QTOF-MS/MS

Anthocyanins were analyzed in positive mode (ESI+) by LC-MS and the profiles of HCADs and other phenolic compounds were analyzed in negative mode (ESI–). The assignment of identity was carried out using PUBCHEM (P), METFRAG (M), Human Metabolome database HMDB (H), and Kyoto encyclopedia of genes and genomes KEEG (K) databases, as well as comparing results with other publications. The level of annotation, in almost all cases, was at least level 2, according to the definition of Sumner et al. [28]. 

The anthocyanins identified in calafate are presented in Table 1. The main anthocyanins detected in this study were the same as those described by Ruiz et al. [8]; however, minor anthocyanins reported in the cited publication were not detected by us, despite the high sensitivity of the MS system. This could be due to the low concentration of formic acid used in the mobile phase (0.1%), which did not allow the anthocyanin structures to stabilize (between flavylium cation, quinoidal base, carbinol, and chalcone forms) [29]. This nonequilibrium state can also explain the peak widening and the low chromatographic resolution, responsible for a lower sensitivity (chromatogram in Supplementary Material, Figure S1). Additionally, in the same chromatogram, a mass of [M]^+^ 336.1213 *m*/*z* (Signal 14) was found, which was assigned as berberine, an alkaloid previously described in calafate [10]. The fragments of 321.0996 *m*/*z* [C_19_H_15_NO_4_]^+^ and 306.0761 *m*/*z* [C_18_H_12_NO_4_]^+^ are products of demethylations of the berberine structure. The fragment 292.0968 *m*/*z* [C_18_H_12_NO_3_ + 2H]^+^ was obtained from a cross fragmentation in the cyclic ether. Signal 13 was tentatively annotated as protoberberine (jatrorrhizine) with [M^+^] 338.1371 *m*/*z* and fragments 294.1099 *m*/*z* [C_18_H_16_NO_3_]^+^ and 323.1121 *m*/*z* [C_19_H_17_NO_4_]^+^. 

The phenolic compounds profile obtained by negative mode (ESI–) showed mainly HCADs (chromatogram in Supplementary Material, Figure S2). Other compounds assigned as flavonols, chalcone, depsides, and hydrolysable tannins were detected. 3-caffeoylquinic acid and caffeoylglucaric isomers were the main compounds detected, which were in accordance with previous findings [9,12,30]. 

Additionally, we assigned identities for the following compounds that have not been detected before in calafate: caffeoylisocitrate isomers (signals 23, 26, 28, 41) that showed a [M-H]^−^ (deprotonated molecule) of 353.0521 *m*/*z* and the following fragments: 191.0197 *m*/*z* [C_6_H_7_O_7_]^−^ corresponding to an isocitrate group, 179.0350 *m*/*z* [C_9_H_7_O_4_]^−^ associated with caffeic acid, and 161.0244 *m*/*z* [C_9_H_6_O_3_-H]^−^ corresponding to caffeoyl group moiety. *O*-feruloylgalactaric acid (signal 29) was tentatively assigned by its ion [M-H]^−^ of 385.0758 *m*/*z* with fragments 191.0197 *m*/*z* [C_6_H_8_O_7_-H]^−^ (galactaroyl), 223.0459 *m*/*z* [C_7_H_11_O_8_]^−^ (methylated isocitrate), and 179.0350 *m*/*z* [C_9_H_6_O_4_+H]^−^ (caffeic acid). All these signals showed a maximum absorbance at 329 nm, which is characteristic of HCADs. Syringic acid glucuronide, a hydrolyzable tannin (signal 19), showed a pseudomolecular ion of [M-H_2_O-H]^−^ 355.0660 *m*/*z*, and a fragment of 147.0299 *m*/*z* [C_5_H_7_O_5_]^−^ (glucuronide group moiety). 3-Hydroxy-4-metoxy-5-(3,4,5-trihydroxybenzoyloxy)benzoic acid (signal 22), known as depside or depsidone, was characterized by [M-H]^−^ 335.0409 *m*/*z* and a fragment of 183.0299 *m*/*z* [C_8_H_7_O_5_]^−^ corresponding to 3-hydroxy-4-metoxybenzoic acid by rupture of an ester bond. Benzyl *O*-(arabinofuranosyl-glucoside) (signal 30), an *O*-glycosyl compound, was characterized by a [M-H]^−^ of 401.1425 *m*/*z* [31] and a fragment of 161.0455 *m*/*z* [C_6_H_9_O_5_]^−^ (a fragment from the glucoside group modified in the oxygen of ether bond). Coumaroyl hexoside (signals 27 and 33) with [M-H]^−^ 325.0920 *m*/*z* and fragments 119.0502 *m*/*z* [C_8_H_7_O]^−^ (coumaric acid decarboxylation), 163.0401 *m*/*z* [C_9_H_7_O_3_]^−^ (coumaric acid) [31], and 145.0295 *m*/*z* [C_9_H_7_O_2_-2H]^−^ (coumaroyl group) was identified. Sinapinic acid-O-glucuronide isomer (signal 37) corresponded to HCADs with an ion [M-H]^−^ of 399.0911 *m*/*z* and fragments 161.0244 *m*/*z* [C_9_H_5_O_3_]^−^ (demethoxylated sinapinic acid), 191.0197 *m*/*z* [C_10_H_7_O_4_]^−^ (sinapinic acid moiety), and 173.0092 *m*/*z* [C_10_H_5_O_3_]^−^, corresponding to [C_10_H_7_O_4_-H_2_O]^−^. Isorhamnetin 3-*O*-(6-*O*-rhamnosylglucoside (signal 46) corresponded to a flavonol characterized by λ_max_ 355 nm, a [M-H]^−^ 623.1592 *m*/*z*, fragments of 315.0510 *m*/*z* [C_16_H_11_O_7_]^−^ (isorhamnetin aglycone) [31], and 300.0276 *m*/*z* [C_15_H_8_O_7_]^−^ (demethylated aglycone). Finally, we found 2′,3,4,4′,6′-pentahydroxychalcone 4′-*O*-glucoside (signal 35), a product of the equilibrium of cyanidin-glucoside flavylium-hemiketal-chalcone. This compound was explained by the ion [M-H]^−^ 449.1064 *m*/*z* and fragment 259.0612 *m*/*z* [C_14_H_11_O_5_]^−^ (aglycone moiety). In total, we found 53 compounds, 17 of them detected in calafate fruit for first the time.

### 3.2. Fatty Acid Profile of Calafate Berry by GC-MS

In this study, we were the first to describe the fatty acid profile of a calafate berry. Table 2 displays the main fatty acid methyl ester (FAME) detected, and the chromatogram is shown as Supplementary Material (Figure S3).

Seven fatty acids were detected in calafate, two of which were essential poly-unsaturated fatty acids, linoleic acid (LA) (12.7%) and linolenic acid (ALA) (15.6%), and monounsaturated fatty acids (oleic and erucic acids, 19.9%). Several studies suggest a favorable relationship between cardiovascular health and unsaturated fatty acid consumption. Regular intake of these fatty acids has been associated with a lower content of total plasma cholesterol and LDL levels, as well as a reduction of atherosclerosis and a decreased incidence of cardiovascular disease [32]. A study carried out with HUVECs demonstrated that the incubation of cells with an Ω 3 fatty acid (DHA) decreased the expression of adhesion molecules, such as vascular cell adhesion molecule-1 (VCAM-1), and the generation of nicotinamide adenine dinucleotide phosphate (NADPH) oxidase-induced ROS in response to IL-1β [33]. Furthermore, it has been demonstrated that LA: 18:2, n-6 and ALA: 18:3, n-3 decrease ROS levels in cells exposed to high glucose [34]. Considering these antecedents and the fatty acid profile, although low in total concentration, these properties, together with the other detected compounds, can explain the beneficial effects of calafate consumption in the context of CVD.

### 3.3. Metal Contents in Calafate Berry by TXRF

The metal profile of calafate fruit has not been previously described. TXRF is a surface elemental analysis technique often used for ultra-trace analysis [35]. The methanolic calafate extract was analyzed in order to identify and quantify the main metals. Corresponding X-ray fluorescence spectra are shown in the Supplementary Material (Figure S4).

The metals detected in calafate were K, Ca, P, S, Mn, Cu, Pb, and Zn. Heavy metals, such as As and Se, were not detected; moreover, Al was under the limit of detection (LOD). The estimated concentrations are listed in Table 3. Due to cross contamination in the TXRF system, Fe was not measured using this technique and its quantitation was carried out by atomic absorption spectroscopy (Supplementary Material). However, Fe had a concentration below the quantification limit of the method (<0.71 μg/g).

Zn, Cu, and Mn play important roles in the regulation of oxidative stress. They form complexes with superoxide dismutase (SOD) that transform superoxide anions into H_2_O_2_, which is subsequently transformed into water and oxygen by catalase. Mn is the main activator of enzymes involved in the antioxidant protection of the human body, such as mitochondrial SOD [20].

Interestingly, when comparing Mn concentration in calafate to that in other fruits, such as grapes or raspberries, calafate showed higher levels. In grape varieties, concentrations range from 0.0004 to 0.0006 mg/g (as fresh weight), while raspberries have between 0.015 and 0.041 mg/g (dry weight) [36,37]. Considering Mn consumption has been associated with beneficial effects, this finding also contributes to the understanding of the potential benefits of calafate fruit in prevention of CVD.

Finally, it is important to highlight that the estimated concentration of Cu and Pb was below the maximum limit allowed in food (0.01 and 0.002 mg/g, respectively) (http://www.diariooficial.interior.gob.cl/media/2013/12/17/do-20131217.pdf). This consideration is important in a product for human consumption.

### 3.4. Concentration of Main Compounds in the Extracts

The full characterization of calafate fruit was carried out using methanol as an extraction solvent, as previously described [8,11,12]. However, considering the cellular toxicity of this solvent, we also evaluated an ethanolic extract obtained under the same conditions as the methanolic extract.

The ethanolic extract showed a similar profile of compounds as that observed for the methanolic extract, with minor differences (see Table 1). The comparative results from the quantitative analyses for the main compounds are presented in the Supplementary Material (Tables S1 and S2). The results were expressed as μmol/mL for phenolic compounds and mg/mL for fatty acids and showed no significant difference for the main anthocyanins, HCADs, or fatty acid contents (*p* > 0.05), which indicates a similar capacity for extraction. Furthermore, total concentration of anthocyanins, HCADs, and fatty acids did not show a significant difference either (*p* > 0.05) (Table 4). However, the observed anthocyanin concentration was lower than previously reported [8], but this can be explained by different edaphoclimatic conditions [8]. The important figures of the quantitative method are included in the Supplementary Material (Table S3).

### 3.5. Antioxidant Capacity of Calafate Extracts

The antioxidant capacity of methanolic and ethanolic extracts was determined by TEAC_ABTS_, TEAC_CUPRAC_, TEAC_ORAC_, and Folin–Ciocalteu (Table 4), which was a complementary screening battery to obtain a global view of the antioxidant potential of the extracts. The results showed high antioxidant capacity for both extracts, with no significant differences between them. The concentration of metals also showed no differences between extracts (data not shown). Metals can have an important role because free ions catalyze Fenton-type reactions, which reduce antioxidant capacity. However, Zúñiga et al. demonstrated that Fe and Mn concentrations may increase antioxidant capacity by 11.0% and 8.9%, respectively (results obtained for both free and complexed metals) [38]. Calafate extracts contained 6.0 µg/g FW of Mn but an undetected concentration of Fe. The contribution of Mn to the high antioxidant capacity of this fruit is unknown, as such it is necessary to perform further studies in order to better understand the effects of these metals on the antioxidant capacity of calafate, which could be through interactions with polyphenols or enzymes.

### 3.6. Calafate Extracts Reduced ROS Production Caused by H_2_O_2_ in HUVECs

Despite the high antioxidant capacity of calafate extracts demonstrated by chemical methods, these results do not necessarily correlate with antioxidant effects in biological systems. To evaluate the potential of calafate extracts on oxidative stress, which is an important factor involved in the development of CVD, we assessed intracellular ROS production using HUVECs. The cells were preincubated with the extracts and then exposed to H_2_O_2_, an oxidizing agent that generates oxidative stress in cells [39].

In a first set of experiments, we evaluated whether calafate extracts had any effect on cell viability. This is important since cell viability can interfere with further ROS quantification. As shown in the Supplementary Material (Figure S5), neither methanolic nor ethanolic extract dilutions affected cell viability after 48 h of incubation (*p* > 0.05). In contrast, cells exposed to PBS for 48 h (positive control for cell lysis by serum deprivation) showed a significant reduction of cell viability (approximately 96%).

**Figure 1:**
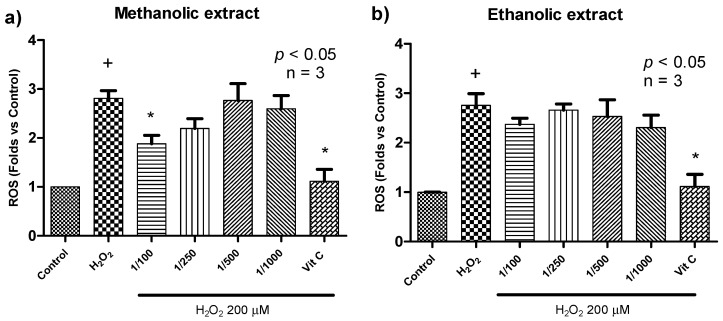
Protective effect of calafate extract on reactive oxygen species (ROS) production upon H_2_O_2_ stimulation. (**a**) methanolic extract diluted at 1/100 (2 mg FW/mL), 1/250 (0.8 mg FW/mL), 1/500 (0.4 mg FW/mL), and 1/1000 (0.2 mg FW/mL). (**b**) Ethanolic extract diluted at 1/100 (2 mg FW/mL), 1/250 (0.8 mg FW/mL), 1/500 (0.4 mg FW/mL), and 1/1000 (0.2 mgFW/mL). Intracellular ROS levels were determined by H_2_DCF-DA fluorescence (10 µM) (λex 485 nm; λem 540 nm) in cells previously incubated with extract and then exposed to H_2_O_2_. Vitamin C (250 µg/mL) was used as the antioxidant control. Data are presented as the mean ± standard error values. + *p* < 0.05 vs. control (negative control). * *p* < 0.05 vs. H_2_O_2_ control (positive control).

Since calafate extracts did not change cell viability, we next evaluated the antioxidant effect of calafate extracts on cells exposed to H_2_O_2_. In this model, we assessed the ability of calafate to prevent ROS production since cells were preincubated with extracts for 24 h, and then, they were removed from culture before the addition of H_2_O_2_. As determined with the H_2_DCF-DA probe (Figure 1), 200 µM H_2_O_2_ induced a 1.8-fold increase in ROS production when compared with nontreated cells (negative control) (*p* < 0.05). The addition of vitamin C together with H_2_O_2_ restored radical production to the basal levels, which is due to the ability of ascorbic ion to react with radicals, producing the ascorbyl radical [20].

In these experiments, the oxidative effect of H_2_O_2_ was reduced by 51% in cells preincubated with the methanolic calafate extract at a dilution of 1/100 (equivalent to 2.22 nmol of anthocyanins and 0.035 nmol of HCAD) (*p* < 0.05) (Figure 1a). As extract dilution increased, the antioxidant effect was reduced and was no longer detected at a dilution of 1/500. For the ethanolic extract, this antioxidant effect was minor (approximately 22% reduction for 1/100 dilution) and did not reach statistical significance (Figure 1b). Due to the lack of a significant difference in the phenolic composition and fatty acids content of the ethanolic and methanolic extracts (Table 4), we hypothesize that minor changes in other compounds may be relevant for the protective effect observed.

It is important to note that the antioxidant protection exerted by the methanolic extract was only detected after long incubations (24 h), since experiments performed with shorter periods (4 h) did not reduce oxidative stress (data not shown). This suggests that the calafate extract induces cell responses, resulting in higher expression/activity of molecules/enzymes involved in the protection against oxidative stress, such as reduced glutathione (GSH) and superoxide dismutase (SOD). This is consistent with findings reported for delphinidin (the main anthocyanin found in calafate extract), which is able to protect SOD activity in HUVECs treated with an oxidant agent for 24 h. In the absence of delphinidin, SOD activity is significantly attenuated by oxidative stress [40]. Moreover, the antioxidant properties of calafate could be related to the effect of metals on the activity of some antioxidant enzymes, as mentioned above. Additionally, despite the low concentration of fatty acids found in calafate extracts, they could still contribute to this antioxidant effect, as reported by Jiang et al., where ALA and LA reduced ROS in HK-2 cells treated with a high glucose concentration [34].

### 3.7. Calafate Extract Reduced Lipidic Peroxidation Caused by CuSO_4_ in Human LDL

**Figure 2:**
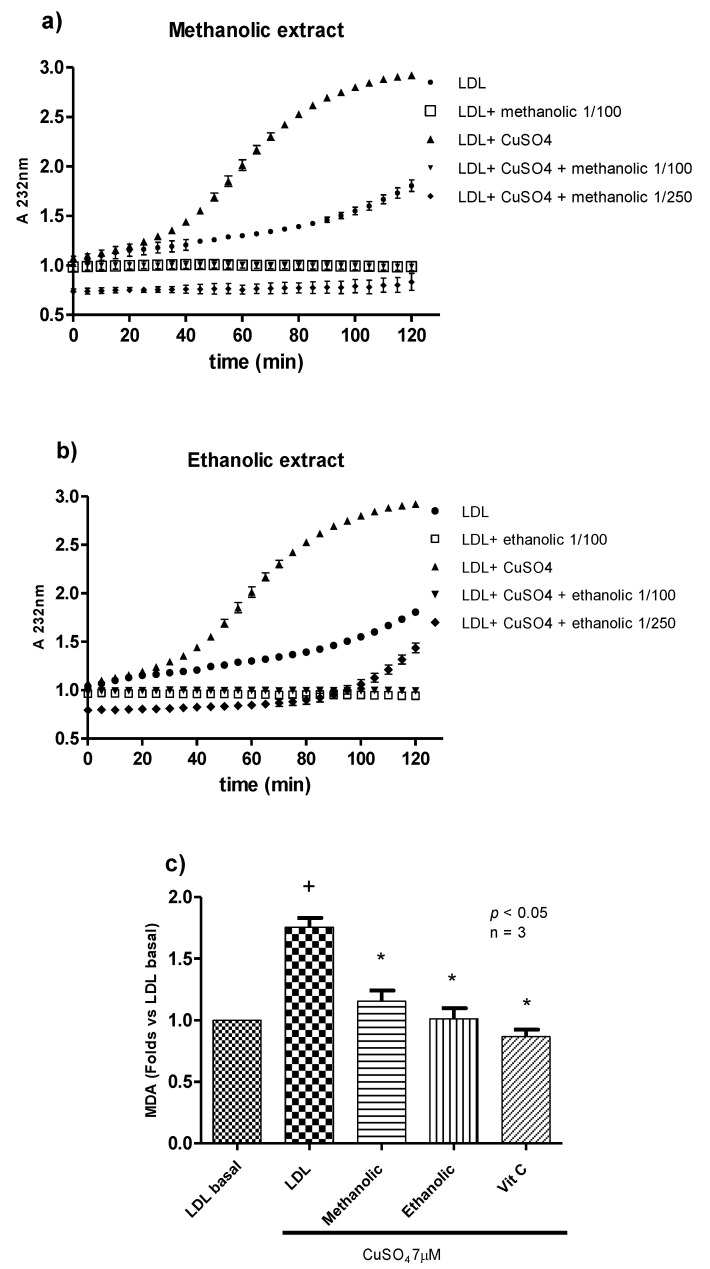
Effect of calafate extract on lipidic peroxidation of human low density lipoprotein (LDL). (**a**) Kinetic curve of LDL oxidation with or without methanolic extract (dilution 1/100 and 1/250). (**b**) Kinetic curve of LDL oxidation with or without ethanolic extract (dilution 1/100 and 1/250). Figures a and b are representative of three LDL assays obtained from different volunteers. (**c**) MDA production in relation to LDL basal control (methanolic and ethanolic extract dilution at 1/100). Data are presented as the mean ± standard error values. Vitamin C (250 µg/mL) was used as the antioxidant control. + *p* < 0.05 vs. LDL basal (negative control). * *p* < 0.05 vs. LDL CuSO_4_ control (positive control).

Oxidative stress is closely related to LDL oxidation and endothelial dysfunction, two important factors contributing to atherosclerosis and CVD [5]. Thus, we evaluated the effect of calafate extracts on lipidic peroxidation of LDL. In Figure 2, the kinetics of human LDL oxidation are presented, as determined by intermediate oxidation products (conjugated dienes measured at 232 nm).

The results showed that native LDL is gradually oxidized by incubation at 37 °C (autoxidation), and after 120 min, conjugated dienes increased by approximately 50%. This reaction speed is rapidly increased with CuSO_4_, as oxidation products began to increase after 40 min of incubation (while LDL without CuSO_4_ was not oxidized yet). They reached the plateau after 90 min, with a 3-fold increase in absorbance, compared to time 0. The methanolic extract inhibited LDL oxidation induced by CuSO_4_ at dilutions of 1/100 (2 mg FW/mL) and 1/250 (0.8 mg FW/mL) throughout the incubation period (Figure 2a). Interestingly, the methanolic extract also inhibited the auto-oxidation of LDL, since it was able to maintain the absorbance at the same values as at time 0, while native LDL (without CuSO_4_) absorbance was increased at the end of the incubation period. Regarding the ethanolic extract, dilutions 1/100 (2 mg FW/mL) and 1/250 (0.8 mg FW/mL) produced the same effect as the methanolic extract by completely inhibiting LDL oxidation (Figure 2b). However, at the end of the incubation, dilution 1/250 began to increase absorbance in the condition with CuSO_4_ to the level of native LDL, suggesting the consumption of antioxidant molecules by this reaction. These results are in concordance with ROS production experiments in which the ethanolic extract showed a lower protective effect than the methanolic extract and confirm that other compounds present in calafate, other than phenolic compounds, can contribute to the antioxidant effect.

Additionally, we measured MDA in LDL, as a final product of the oxidation process. In these experiments, MDA was assessed after 80 min of oxidation. Under these conditions, calafate extracts reduced MDA produced by CuSO_4_ to the levels found in native LDL (Figure 2c). This effect was comparable to the effect obtained with vitamin C (*p* < 0.05). This is an interesting finding attributed to the total compounds present in the extracts, which are responsible for total antioxidant capacity.

The effect of the extracts against LDL oxidation may be produced by the ability of polyphenols (flavonoids) to eliminate free radicals or chelate metal ions, such as copper in this case (oxidant agent) [41]. However, to establish which compounds are responsible for these effects, it is necessary to carry out complementary experiments.

In a biological context, LDL oxidation plays a very important role in cardiovascular disease [15]. Oxidized LDL is atherogenic, as it causes direct oxidative damage to endothelial cells, promotes the inflammatory response, and accumulates in the intima of blood vessels, where it is recognized and endocytosed by macrophages, promoting their transformation to foam cells, one of the main components of the atherosclerotic plaque [1]. Compounds that can prevent oxidative processes have been demonstrated to attenuate or prevent the development of atherosclerosis. Our findings suggest that consumption of calafate fruit could have a potential anti-atherosclerotic effect, which can be mainly attributed to the phenolic compounds composition and additionally to the metals and poly-unsaturated fatty acids, which may also have an important role in the beneficial effect of calafate.

## 4. Conclusions

Calafate fruit has a broad profile of bioactive compounds, mainly including phenolic compounds, which compose more than 53 chromatographic signals tentatively identified (anthocyanins, HCADs, flavonols, and other phenolics and alkaloids such as berberine), fatty acids (mainly linolenic and linoleic acids), and certain metals (Mn, Cu, and Zn, among others). This composition constitutes a product with remarkable nutraceutical characteristics for human consumption. Calafate extracts have a high chemical antioxidant capacity, can decrease ROS production in a cellular model of endothelium, and protect LDL against oxidation, which is one of the main factors responsible for the progression of atherosclerosis. Finally, based on our results, we propose that the consumption of calafate can play a role in the prevention of cardiovascular disease, the main cause of death in Chile and around the world; however, in order to confirm the effect of calafate in preventing CVD, further studies must be carried out, including animal, preclinical and clinical trials.

## Figures and Tables

**Table 1:** Metabolic profile of calafate obtained by UHPLC-DAD-QTOF in positive and negative ionization modes.

Nº Peak	t_R_^e^ (min)	λ ^f^ (nm)	Molecular Formula	[M]^+^ or [M-H]^−^	ESI	Fragments ^g^	Error (ppm)	m_σ_^h^	Annotation	Ref and/or DB
1 ^c^	7.6	276; 523	C_27_H_31_O_17_^+^	627.1527	+	303.0499; 229.0495; 465.1028	4.6	4.2	delphinidin-3,7-*β-O*-diglucoside	[8,9,18] M, K
2 ^b^	8.1	276; 524	C_28_H_33_O_17_^+^	641.1678	+	317.0656	5.4	5.5	petunidin- 3,7-*β-O*- diglucoside	[8,9,18]
3 ^b^	8.7	-	C_29_H_35_O_17_^+^	655.1840	+	331.0812	4.3	18.1	malvidin- 3,7-*β-O-*diglucoside	[8,9,18]
4 ^c^	9.8	277; 525	C_27_H_31_O_16_^+^	611.1582	+	303.0499; 229.0495	4.1	4.3	delphinidin 3-rutinoside	[8,11], M, K
5 ^c^	9.9	276; 525	C_21_H_21_O_12_^+^	465.0989	+	303.0499; 229.0495	8.4	5.6	delphinidin 3-glucoside	[8,11,14,18] M, K
6 ^c^	12.0	280; 519	C_27_H_31_O_15_^+^	595.1646	+	287.0550; 242.0574	1.9	14.0	cyanidin 3-rutinoside	[8,11,14], M, K
7 ^c^	12.1	279; 519	C_21_H_21_O_11_^+^	449.1059	+	287.0550; 115.0542	4.3	0.9	cyanidin 3-glucoside	[8,11,14,18], M, K
8 ^c^	12.1	279; 519	C_22_H_23_O_12_^+^	479.1170	+	317.0656; 217.0495; 302.0421; 245.0444; 174.0311; 192.0417; 229.0495; 274.0472	3.0	6.0	petunidin 3-glucoside	[8,11,14,18], H
9 ^b^	12.3	281; 524	C_28_H_33_O_16_^+^	625.1764	+	317.0656	0.1	4.9	petunidin 3-rutinoside	[8,11,14]
10 ^b^	14.7	277; 529	C_29_H_35_O_16_^+^	639.1923	+	331.0812	−0.5	3.1	malvinidin 3-rutinoside	[8,11]
11 ^c^	14.7	278; 529	C_22_H_23_O_11_^+^	463.1228	+	301.0707; 286.0472; 229.0495; 187.0390; 213.0546; 203.0339	1.5	4.0	peonidin 3-glucoside	[8,11,14,18], M, K
12 ^c^	14.8	277; 529	C_23_H_25_O_12_^+^	493.1338	+	331.0812; 287.0550; 316.0578; 242.0574; 245.0444; 217.0495	0.6	5.3	malvidin 3-glucoside	[8,11,18], M, K
13 ^b^	28.3	221; 251; 350	C_20_H_20_NO_4_^+^	338.1371	+	294.1099; 323.1191; 280.0955	5.5		jatrorrhizine	[10]
14 ^c^	32.4	221; 251; 350	C_20_H_18_NO_4_^+^	336.1213	+	292.0968; 306.0761; 321.0996; 278.0812	5.3	16.7	berberine	[10], M, K
15 ^c^	11.3	326	C_15_H_16_O_11_	371.0606	-	209.0303; 191.0197; 135.0452; 179.0350; 147.0299	3.8	4.3	3- or 4-trans- caffeoyl-glucaric acid	[9,12,30], M, K
16 ^c^	12.1	326	C_15_H_16_O_11_	371.0619	-	209.0303; 191.0197; 135.0452; 147.0299; 179.0350; 129.0193; 193.0506	0.1	9.0	3- or 4-trans- caffeoyl-glucaric acid	[9,12,30], M, K
17 ^c^	12.7	325	C_15_H_16_O_11_	371.0619	-	209.0303; 163.0401; 191.0197; 119.0502; 135.0452; 147.0299; 179.0350	0.1	10.8	2- or 5-trans-caffeoyl-glucaric acid	[9,12,30], M, K
18 ^c^	13.4	-	C_16_H_18_O_9_	353.0889	-	191.0561; 135.0452; 179.0350; 93.0346; 161.0244	−3.0	18.3	caffeoylquinic acid isomer	[12,30,31], M, K
19 ^a^	13.7 ^A^	-	C_15_H_18_O_11_	355.0660 [M-H_2_O-H]^−^	-	209.0303; 147.0299; 239.0561; 119.0502	3.0	16.9	syringic acid glucuronide	H
20 ^c^	13.8	-	C_15_H_16_O_11_	371.0619	-	209.0303; 191.0197; 135.0452; 147.0299; 115.0037; 173.0092	0.1	9.4	caffeoyl glucaric isomer	[12,30], M, K
21 ^c^	14.1 ^B^	-	C_15_H_16_O_11_	371.0626	-	209.0303; 191.0197; 135.0452; 173.0092	−1.7	2.6	caffeoyl glucaric isomer	[12,30], M, K
22 ^a^	14.7	-	C_15_H_12_O_9_	335.0409	-	183.0299; 177.0193	−0.2	4	3-hydroxy-4-metoxy-5-(3,4,5-trihydroxybenzoyloxy) benzoic acid	H
23 ^a^	14.9	327	C_15_H_14_O_10_	353.0521	-	191.0197; 135.0452; 147.0299; 179.0350; 161.0244	−2.0	18.4	caffeoyl isocitrate isomer	P, M, K
24	15.6	-	C_13_H_24_O_9_	323.1345	-	113.0244; 179.0561; 101.0244; 161.0455	0.8	2.4	unidentified	
25 ^d^	16.0	326	C_16_H_18_O_9_	353.0866	-	191.0561; 127.0401; 161.0244; 93.0346	3.3	1.5	3-caffeoyl quinic acid	[12,30,31], M, K
26 ^a^	16.6	-	C_15_H_14_O_10_	353.0506	-	161.0244; 191.0197; 147.0299; 135.0452; 117.0346	2.4	4.2	caffeoyl lisocitrate isomer	P, M, K
27 ^c^	16.8	-	C_15_H_18_O_8_	325.0920	-	119.0502; 163.0401	1.6	2.7	coumaroyl hexoside	[31], M, K
28 ^a^	17.5	329	C_15_H_14_O_10_	353.0491	-	191.0197; 147.0299; 135.0452; 179.0350	6.5	12.0	caffeoyl isocitrate isomer	P, M, K
29 ^a^	17.5 ^A^	329	C_16_H_18_O_11_	385.0758	-	191.0197; 223.0459; 179.0350	4.8	10.9	*O*-feruloyl galactaric acid	M, K
30^c^	18.1	-	C_18_H_26_O_10_	401.1425	-	161.0455	7.1	4.6	benzyl *O*-(arabino furanosyl-glucoside)	[31], H
31 ^b^	18.3	-	C_24_H_22_O_14_	533.0911	-	209.0303; 191.0197	4.9	9.0	dicaffeoyl glucaric acid isomer	[12,30]
32 ^b^	18.6	-	C_24_H_22_O_14_	533.0896	-	209.0303; 191.0197	7.6	4,1	dicaffeoyl glucaric acid isomer	[12,30]
33 ^c^	18.7	-	C_15_H_18_O_8_	325.0904	-	145.0295; 117.0346	7.6	15.5	coumaroyl hexoside	[31], M, K
34 ^c^	19.0	-	C_16_H_18_O_9_	353.0851	-	191.0561; 161.0244; 93.0346; 127.0401; 133.0295	7.6	19.4	caffeoylquinic acid isomer	[12,30,31], M, K
35 ^a^	20.1	-	C_21_H_22_O_11_	449.1064	-	259.0612; 191.0561; 97.0295	5.6	17.2	2′,3,4,4′,6′-Pentahydroxychalcone 4′-*O*-glucoside	M, K
36 ^c^	20.4	-	C_16_H_18_O_8_	337.0899	-	191.0561; 119.0502; 93.0346; 163.0401; 145.0295; 155.0350	8.9	5.1	coumaroyl quinic acid	[12,30,31], M, K
37 ^a^	21.9	-	C_17_H_20_O_11_	399.0911	-	161.0244; 191.0197; 173.0092; 367.0671	5.6	5.1	sinapinic acid-*O*-glucuronide	H
38 ^c^	22.5	354	C_27_H_30_O_16_	609.1435	-	301.0354; 255.0299; 151.0037; 178.9986	4.3	5.7	quercetin-3-rutinoside	[10,18,30,31], M, K
39	23.1	-	C_18_H_32_O_12_	439.1799	-	149.0455; 179.0561; 119.0350; 101.0244; 251.0772	5.0	15.3	unidentified	
40 ^c^	23.4 ^A^	-	C_17_H_20_O_9_	367.1003	-	135.0452; 161.0244; 179.0350; 191.0561; 137.0244; 127.0401; 117.0346	8.6	8.2	feruloylquinic acid isomer	[12,30,31], M, K
41 ^a^	23.4	-	C_15_H_14_O_10_	353.0489	-	135.0452; 129.0193; 179.0350; 161.0244; 173.0092; 219.0299; 151.0401; 335.0409	7.1	15.1	caffeoyl isocitrate isomer	P, M, K
42 ^b^	24.6	355	C_21_H_20_O_12_			271.0248; 301.0354; 255.0299; 151.0037; 216.0428	3.9	7.4	quercetin-3-galactoside	[10]
43 ^d^	24.9	354	C_21_H_20_O_12_	463.0861	-	271.0248; 301.0354; 255.0299; 151.0037; 245.0455; 178.9986	4.5	7.5	quercetin-3-glucoside	[10], M, K
44	25.0	-	C_18_H_32_O_10_	407.1903	-	227.1289; 138.1050	4.7	10.4	Unidentified	
45 ^c^	25.4	-	C_27_H_30_O_15_	593.1499	-	285.0405; 257.0397; 549.1250	2.1	19.1	kaempferol-3-*O*-rutinoside	[30], M, K
46 ^b^	25.8	355	C_28_H_32_O_16_	623.1592	-	315.0510; 300.0276; 287.0561	4.1	2.6	isorhamnetin 3-*O*-(6-*O*-rhamnosyl-glucoside	[31]
47 ^c^	26.5	-	C_24_H_22_O_15_	549.0878	-	505.0972; 271.0248; 301.0295; 301.0354; 255.0299; 151.0037; 178.9986; 187.0401	1.8	13.4	quercetin-3-malonyl galactoside	[10], M, K
48 ^c^	27.1	353	C_24_H_22_O_15_	549.0895	-	505.0988; 255.0299; 463.0882; 178.9986; 151.0037; 283.0248	0.7	10.3	quercetin-3-malonyl glucoside	[10], M, K
49 ^b^	27.8	-	C_22_H_22_O_12_	477.1033	-	243.0299; 271.0248; 257.0455; 286.0483; 300.0276; 215.0350	1.2	9.9	isorhamnetin-3-glucoside	[10,30,31]
50 ^c^	28.5	348	C_21_H_20_O_11_	447.0929	-	271.0248; 301.0354; 255.0299; 151.0037; 245.0397; 178.9986; 109.0295; 121.0295; 135.0088	0.9	2.9	quercetin 3-L-rhamnoside	[10,30,31], M, K
51 ^c^	29.6	-	C_25_H_24_O_12_	515.1194	-	353.0878; 173.0455; 179.0350; 191.0561; 135.0452; 155.0350; 93.0346; 161.0244; 318.0686; 137.0244; 203.0350; 356.0902; 111.0452	0.1	16.0	3,5-dicaffeoyl quinic acid	[12,31], M, K
52 ^b^	30.0	-	C_24_H_24_O_13_	519.1134	-	300.0276; 315.0510; 227.0350; 177.0193; 204.0428; 204.0487	2.0	3.2	isorhamnetin-3-malonyl-hexoside	[10]
53 ^c^	31.7	-	C_21_H_20_O_10_	431.0974		285.0405; 255.0299; 244.0377; 267.0358	2.3	6.4	kaempferol rhamnoside	[10,30,31], M, K

^A^ Compounds detected only in the methanolic extract. ^B^ Compounds detected only in the ethanolic extract. Peaks identified with ^a^ Database (DB): PUBCHEM (P), METFRAG (M), HMDB (H), and KEEG (K), ^b^ bibliography, ^c^ Database plus bibliography, ^d^ Database, bibliography, and commercial standards. ^e^ t_R_: retention time. ^f^ λ: wavelength. ^g^ Higher to lower intensity. ^h^ m_σ_: Bruker parameter. UHPLC-DAD-QTOF: Ultra-Liquid Chromatography with Diode Array Detector, coupled to Quadrupole-Time of Fly Mass Spectrometry.

**Table 2:** Fatty acids profile of calafate fruit (GC-MS).

t_R_ (min)	Formula	Assigned Identity	Mass (Da)	Concentration (mg/g FW ^a^)
19.972	C_15_H_30_O_2_	Methyl tetradecanoate (Myristic acid methyl ester)	242.2	0.16 ± 0.01
22.443	C_17_H_34_O_2_	Hexadecanoic acid, methyl ester	270.3	0.08 ± 0.02
24.708	C_19_H_38_O_2_	Octadecanoic acid, methyl ester	298.3	0.06 ± 0.00
25.507	C_19_H_36_O_2_	9-Octadecenoic acid, methyl ester, (Z) (Oleic acid methyl ester)	296.3	0.04 ± 0.01
26.783	C_19_H_34_O_2_	9,12-Octadecadienoic acid (Z,Z)-, methyl ester (Linoleic acid methyl ester ω6)	294.3	0.07 ± 0.03
28.160	C_19_H_32_O_2_	9,12,15-Octadecatrienoic acid, methyl ester, (Z,Z,Z)- (Linolenic acid methyl ester ω3)	292.2	0.09 ± 0.04
29.473	C_23_H_44_O_2_	13-Docosenoic acid, methyl ester, (Z)- (Erucic acid methyl ester)	352.3	0.08 ± 0.01

^a^ FW: Fresh Weight. Data presented as the mean ± standard error. t_R_: retention time.

**Table 3:** Estimated concentration of metals in calafate berry by TXRF.

Metals	Calafate mg/g FW ^b^
Aluminum	ND
Phosphorus	0.830 ± 0.040
Sulfur	0.610 ± 0.010
Potassium	3.460 ± 0.110
Calcium	0.550 ± 0.020
Manganese	0.006 ± 0.000
Copper	0.006 ± 0.000
Zinc	0.007 ± 0.000
Lead	0.001 ± 0.000
Iron ^a^	<0.00071

^a^ Determined by atomic absorption spectroscopy. ^b^ FW: fresh weight. ND: not detected. TXRF: Total X-ray Fluorescence.

**Table 4:** Total polyphenol concentration and antioxidant capacity in calafate extracts.

Extract	Total Anthocyanins	Total Hydroxycinnamic Acids	Total Fatty Acids	TEAC_ABTS_	TEAC_CUPRAC_	TEAC_ORAC_	Folin–Ciocalteu
	(μmol/mL)	(μmol/mL)	(mg/mL)	(Trolox Equivalents μmol/mL)			(Equi-Valents. Gallic Acid mg/mL)
**Methanolic**	2.22 ± 0.18	0.35 ± 0.01	0.37 ± 0.04	13.74 ± 0.76	21.14 ± 0.28	21.38 ± 0.64	1.90 ± 0.04
**Ethanolic**	2.16 ± 0.07	0.28 ± 0.02	0.51 ± 0.04	10.88 ± 0.72 *	19.36 ± 0.14 *	21.60 ± 0.86	1.60 ± 0.02 *

* *p* < 0.05 vs. methanolic extract. Data presented as the mean ± standard error. Figures of merit are presented as Supplementary Material (Table S3). TEAC_CUPRAC_: Trolox equivalent antioxidant capacity assay by cupric-reducing antioxidant capacity. TEAC_ABTS_: Trolox equivalent antioxidant capacity assay with 2,2′-azino-bis(3-ethylbenzothiazoline-6-sulfonic acid. TEAC_ORAC_: Trolox equivalent antioxidant capacity assay by oxygen radical absorbance capacity.

## Supplementary Materials

The following are available online at https://www.mdpi.com/2076-3921/9/12/1171/s1, Figure S1: Base peak chromatogram ESI+ crude extract calafate (black) and DAD chromatogram to 520 nm (red), Figure S2: Base peak chromatogram ESI– extract previously purify with solid extraction for hydroxycinnamic acid derivatives (red), DAD chromatogram 320 nm (yellow), and DAD chromatogram 360 nm (green), Figure S3: Extracted ion chromatogram (EIC) of fatty acids profile in calafate fruit (81 *m*/*z*; 87 *m*/*z*; 91 *m*/*z*), Figure S4: X-ray fluorescence spectra of calafate fruit. Internal standard Ga 0.5 mg/L Scans 1200 s, Table S1: Comparative concentration of main polyphenolic compounds identified in calafate berry extracts, Table S2: Comparative concentration of FAME identified in calafate berry extracts, Table S3: Figure of merit of the quantitative method, Figure S5: In vitro viability assay determined by MTT method, and Method “Determination of Fe in calafate extract by atomic absorption spectroscopy (AAS)”.
