# Supplementary material for: Berberis microphylla G. Forst (Calafate) Berry Extract Reduces Oxidative Stress and Lipid Peroxidation of Human LDL
Source: Antioxidants (Basel). 2020 Nov 24;9(12):1171. doi: 10.3390/antiox9121171 (PMC7760614; doi:10.3390/antiox9121171)
Supplement: Supplementary file 1 [file antioxidants-09-01171-s001.zip › antioxidants-993668-supplementary.pptx]

## Slide 1
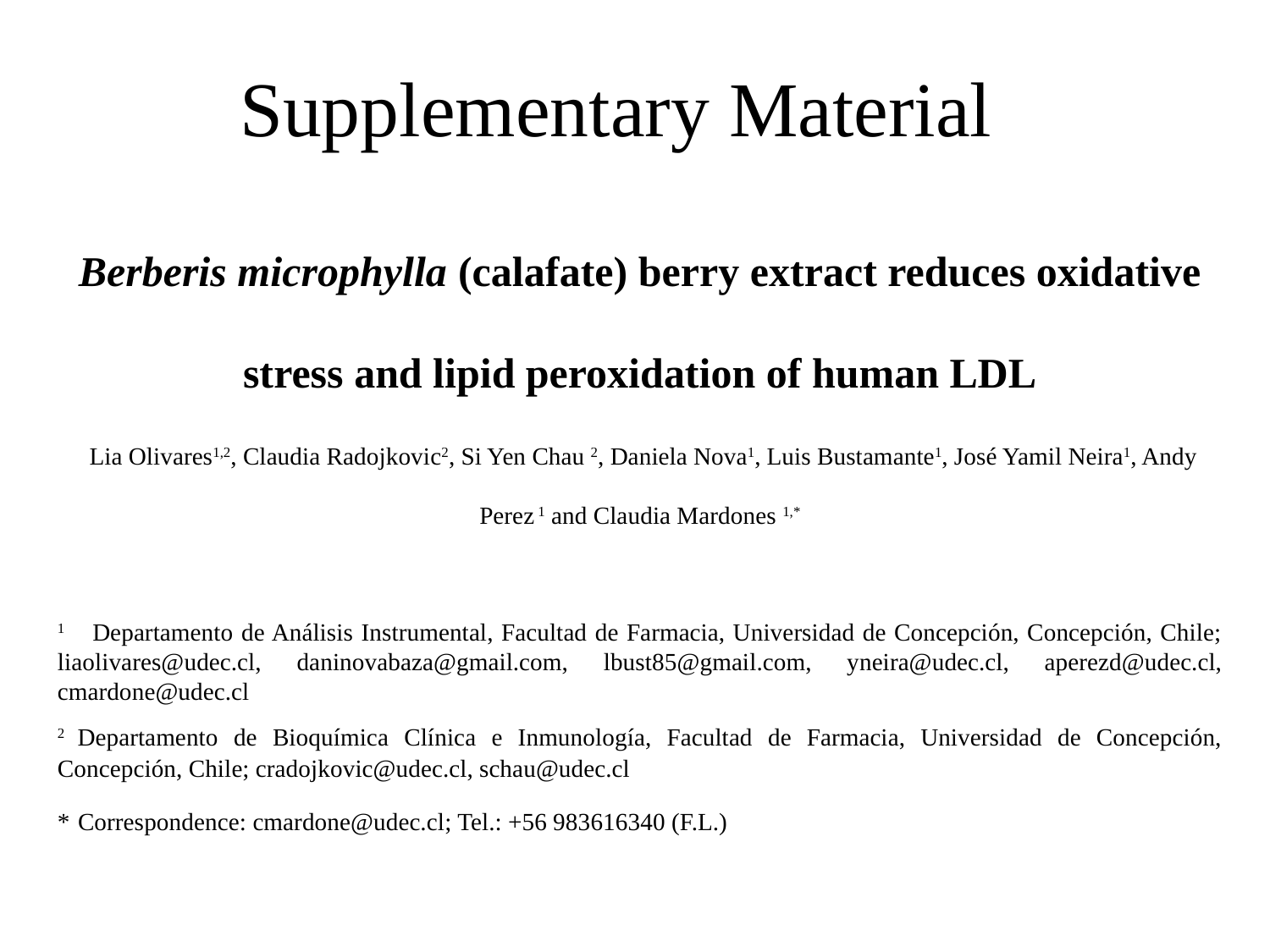

# Supplementary Material
Berberis microphylla (calafate) berry extract reduces oxidative stress and lipid peroxidation of human LDL
 Lia Olivares1,2, Claudia Radojkovic2, Si Yen Chau 2, Daniela Nova1, Luis Bustamante1, José Yamil Neira1, Andy Perez 1 and Claudia Mardones 1,*
1 Departamento de Análisis Instrumental, Facultad de Farmacia, Universidad de Concepción, Concepción, Chile; liaolivares@udec.cl, daninovabaza@gmail.com, lbust85@gmail.com, yneira@udec.cl, aperezd@udec.cl, cmardone@udec.cl
2 Departamento de Bioquímica Clínica e Inmunología, Facultad de Farmacia, Universidad de Concepción, Concepción, Chile; cradojkovic@udec.cl, schau@udec.cl
* Correspondence: cmardone@udec.cl; Tel.: +56 983616340 (F.L.)

## Slide 2
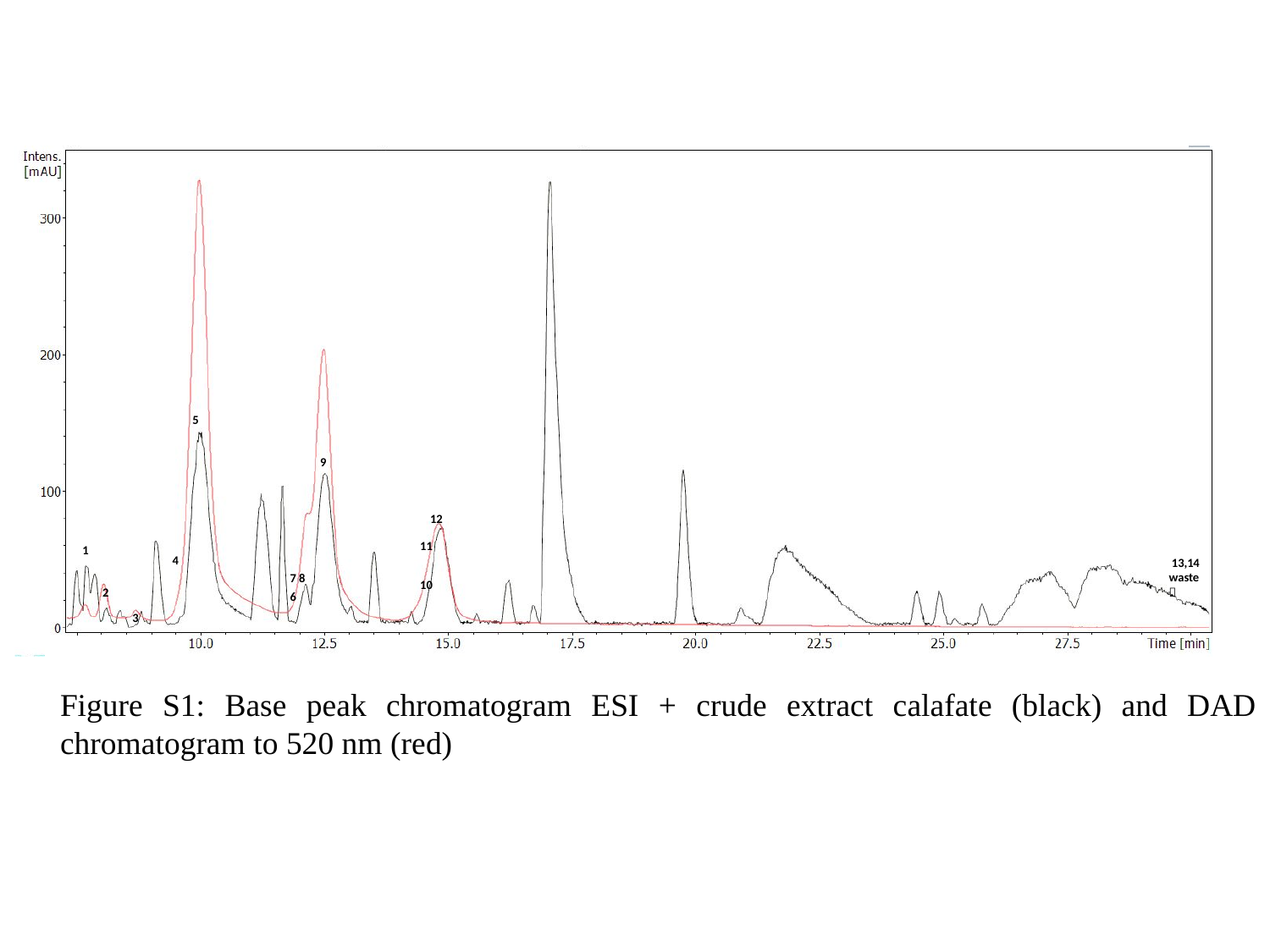

5
 9
 12
 11
1
4
 13,14
waste
 7 8
 10
2
 6
3
Figure S1: Base peak chromatogram ESI + crude extract calafate (black) and DAD chromatogram to 520 nm (red)

## Slide 3
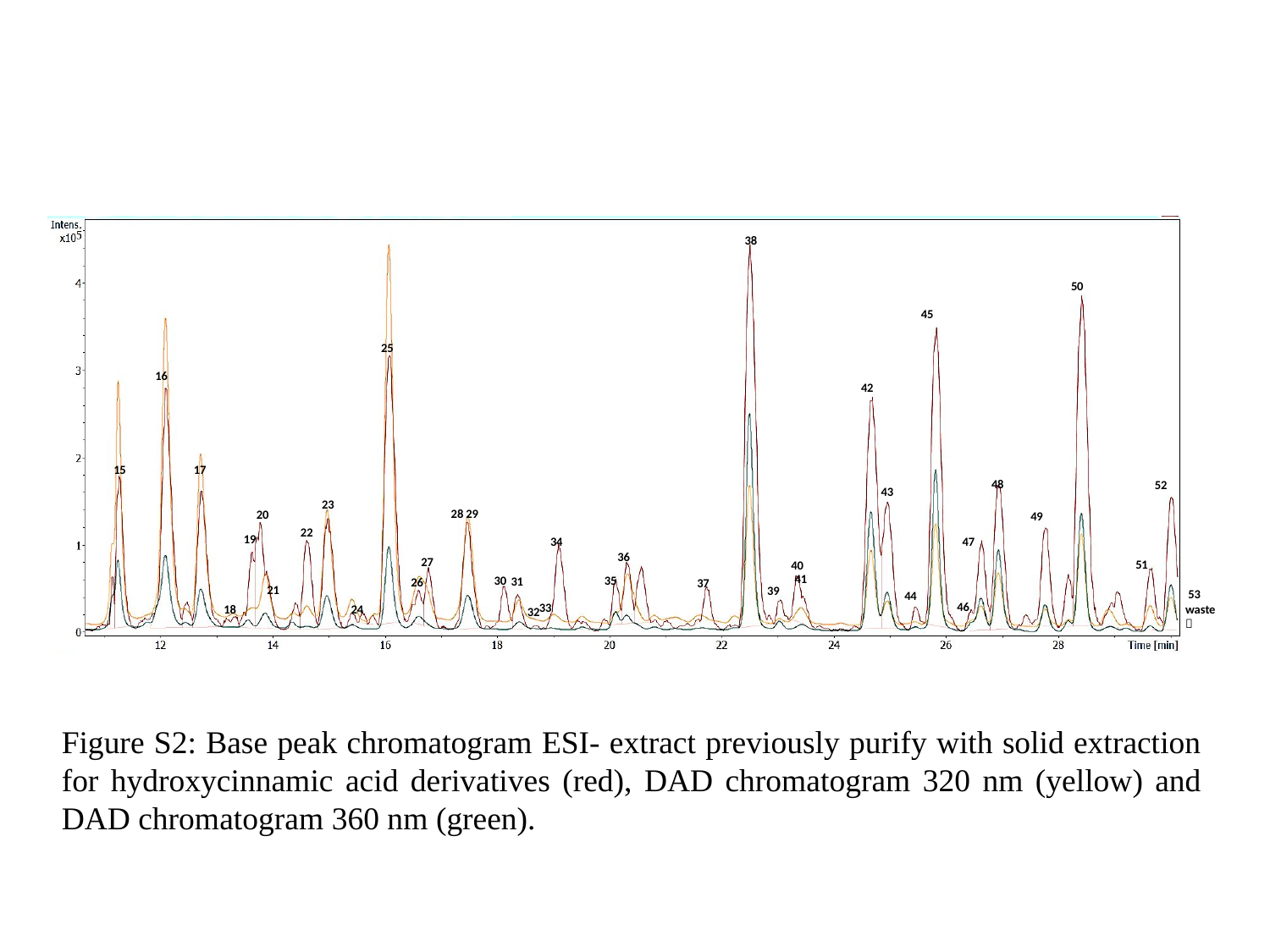

38
50
45
25
 16
42
 15
 17
48
52
43
23
28 29
 20
49
22
 19
34
47
36
27
51
40
41
35
30
31
26
37
21
39
44
46
33
 18
24
32
 53
waste
Figure S2: Base peak chromatogram ESI- extract previously purify with solid extraction for hydroxycinnamic acid derivatives (red), DAD chromatogram 320 nm (yellow) and DAD chromatogram 360 nm (green).

## Slide 4
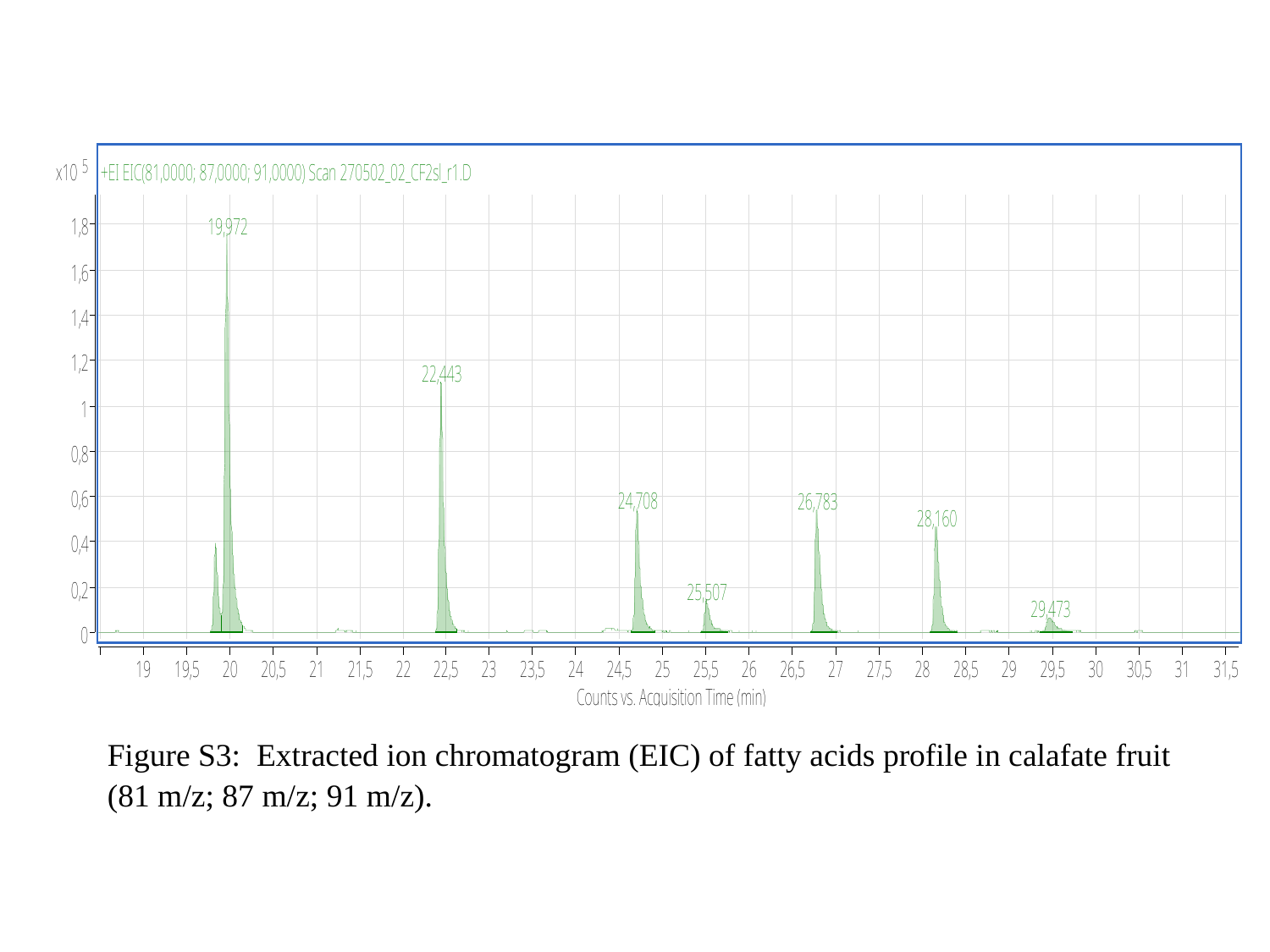

Figure S3: Extracted ion chromatogram (EIC) of fatty acids profile in calafate fruit (81 m/z; 87 m/z; 91 m/z).

## Slide 5
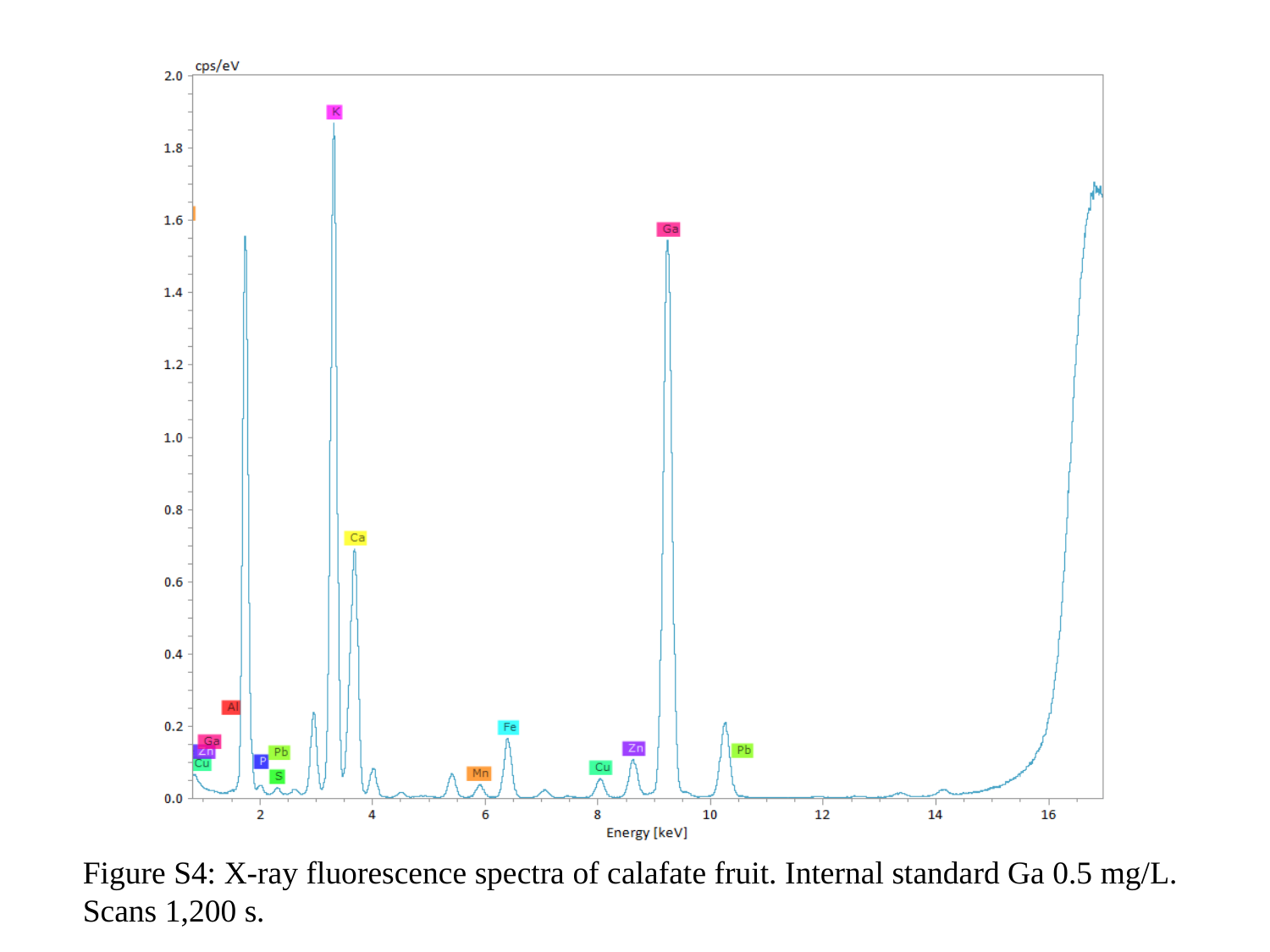

Figure S4: X‐ray fluorescence spectra of calafate fruit. Internal standard Ga 0.5 mg/L.
Scans 1,200 s.

## Slide 6
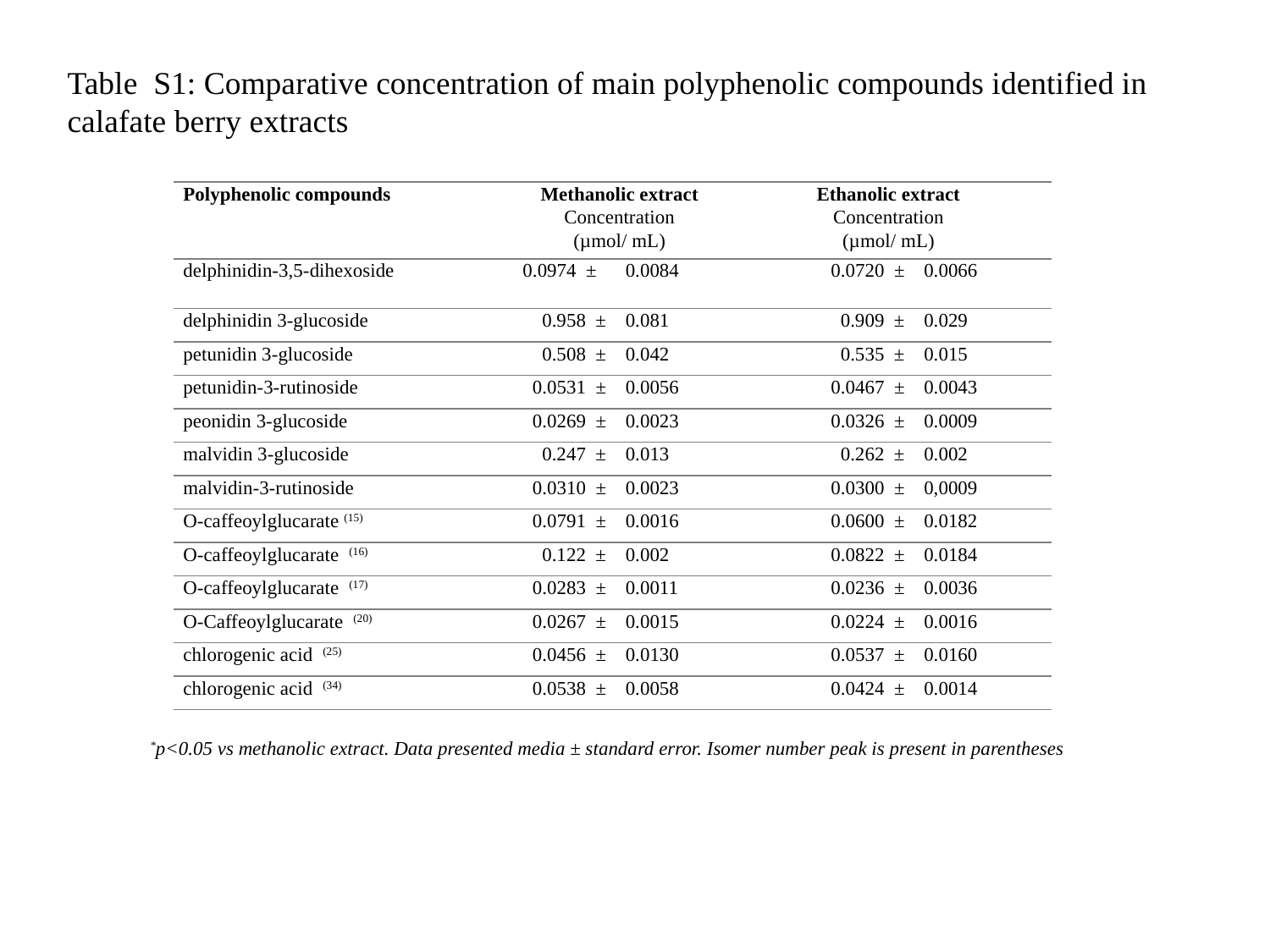

Table S1: Comparative concentration of main polyphenolic compounds identified in calafate berry extracts
| Polyphenolic compounds | Methanolic extract Concentration (µmol/ mL) | | Ethanolic extract Concentration (µmol/ mL) | |
| --- | --- | --- | --- | --- |
| delphinidin-3,5-dihexoside | 0.0974 ± | 0.0084 | 0.0720 ± | 0.0066 |
| delphinidin 3-glucoside | 0.958 ± | 0.081 | 0.909 ± | 0.029 |
| petunidin 3-glucoside | 0.508 ± | 0.042 | 0.535 ± | 0.015 |
| petunidin-3-rutinoside | 0.0531 ± | 0.0056 | 0.0467 ± | 0.0043 |
| peonidin 3-glucoside | 0.0269 ± | 0.0023 | 0.0326 ± | 0.0009 |
| malvidin 3-glucoside | 0.247 ± | 0.013 | 0.262 ± | 0.002 |
| malvidin-3-rutinoside | 0.0310 ± | 0.0023 | 0.0300 ± | 0,0009 |
| O-caffeoylglucarate (15) | 0.0791 ± | 0.0016 | 0.0600 ± | 0.0182 |
| O-caffeoylglucarate (16) | 0.122 ± | 0.002 | 0.0822 ± | 0.0184 |
| O-caffeoylglucarate (17) | 0.0283 ± | 0.0011 | 0.0236 ± | 0.0036 |
| O-Caffeoylglucarate (20) | 0.0267 ± | 0.0015 | 0.0224 ± | 0.0016 |
| chlorogenic acid (25) | 0.0456 ± | 0.0130 | 0.0537 ± | 0.0160 |
| chlorogenic acid (34) | 0.0538 ± | 0.0058 | 0.0424 ± | 0.0014 |
*p<0.05 vs methanolic extract. Data presented media ± standard error. Isomer number peak is present in parentheses

## Slide 7
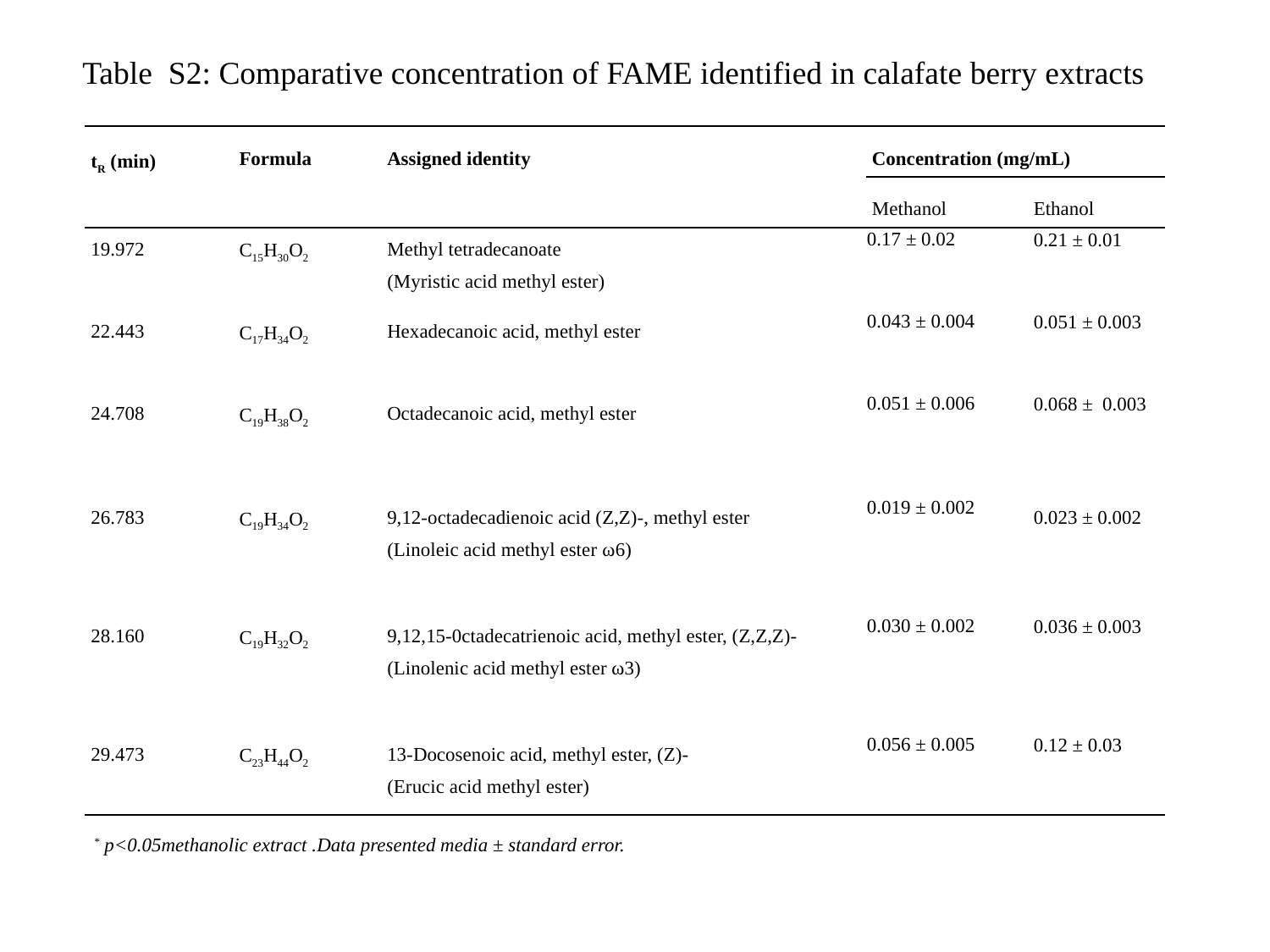

Table S2: Comparative concentration of FAME identified in calafate berry extracts
| tR (min) | Formula | Assigned identity | Concentration (mg/mL) | |
| --- | --- | --- | --- | --- |
| | | | Methanol | Ethanol |
| 19.972 | C15H30O2 | Methyl tetradecanoate (Myristic acid methyl ester) | 0.17 ± 0.02 | 0.21 ± 0.01 |
| 22.443 | C17H34O2 | Hexadecanoic acid, methyl ester | 0.043 ± 0.004 | 0.051 ± 0.003 |
| 24.708 | C19H38O2 | Octadecanoic acid, methyl ester | 0.051 ± 0.006 | 0.068 ±  0.003 |
| 26.783 | C19H34O2 | 9,12-octadecadienoic acid (Z,Z)-, methyl ester (Linoleic acid methyl ester ω6) | 0.019 ± 0.002 | 0.023 ± 0.002 |
| 28.160 | C19H32O2 | 9,12,15-0ctadecatrienoic acid, methyl ester, (Z,Z,Z)- (Linolenic acid methyl ester ω3) | 0.030 ± 0.002 | 0.036 ± 0.003 |
| 29.473 | C23H44O2 | 13-Docosenoic acid, methyl ester, (Z)- (Erucic acid methyl ester) | 0.056 ± 0.005 | 0.12 ± 0.03 |
* p<0.05methanolic extract .Data presented media ± standard error.

## Slide 8
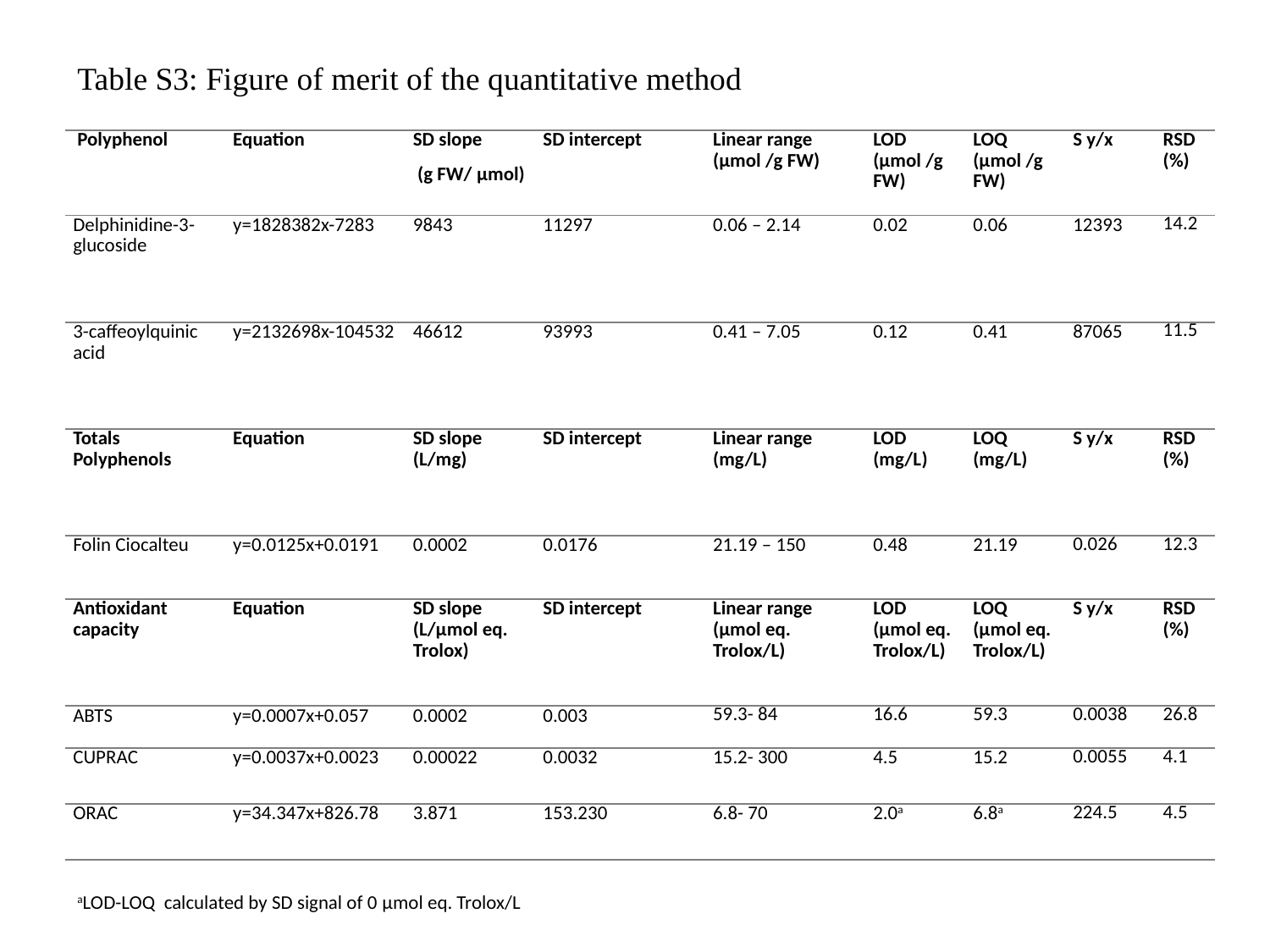

Table S3: Figure of merit of the quantitative method
| Polyphenol | Equation | SD slope (g FW/ μmol) | SD intercept | Linear range (μmol /g FW) | LOD (μmol /g FW) | LOQ (μmol /g FW) | S y/x | RSD (%) |
| --- | --- | --- | --- | --- | --- | --- | --- | --- |
| Delphinidine-3-glucoside | y=1828382x-7283 | 9843 | 11297 | 0.06 – 2.14 | 0.02 | 0.06 | 12393 | 14.2 |
| 3-caffeoylquinic acid | y=2132698x-104532 | 46612 | 93993 | 0.41 – 7.05 | 0.12 | 0.41 | 87065 | 11.5 |
| Totals Polyphenols | Equation | SD slope (L/mg) | SD intercept | Linear range (mg/L) | LOD (mg/L) | LOQ (mg/L) | S y/x | RSD (%) |
| Folin Ciocalteu | y=0.0125x+0.0191 | 0.0002 | 0.0176 | 21.19 – 150 | 0.48 | 21.19 | 0.026 | 12.3 |
| Antioxidant capacity | Equation | SD slope (L/μmol eq. Trolox) | SD intercept | Linear range (μmol eq. Trolox/L) | LOD (μmol eq. Trolox/L) | LOQ (μmol eq. Trolox/L) | S y/x | RSD (%) |
| ABTS | y=0.0007x+0.057 | 0.0002 | 0.003 | 59.3- 84 | 16.6 | 59.3 | 0.0038 | 26.8 |
| CUPRAC | y=0.0037x+0.0023 | 0.00022 | 0.0032 | 15.2- 300 | 4.5 | 15.2 | 0.0055 | 4.1 |
| ORAC | y=34.347x+826.78 | 3.871 | 153.230 | 6.8- 70 | 2.0a | 6.8a | 224.5 | 4.5 |
aLOD-LOQ calculated by SD signal of 0 μmol eq. Trolox/L

## Slide 9
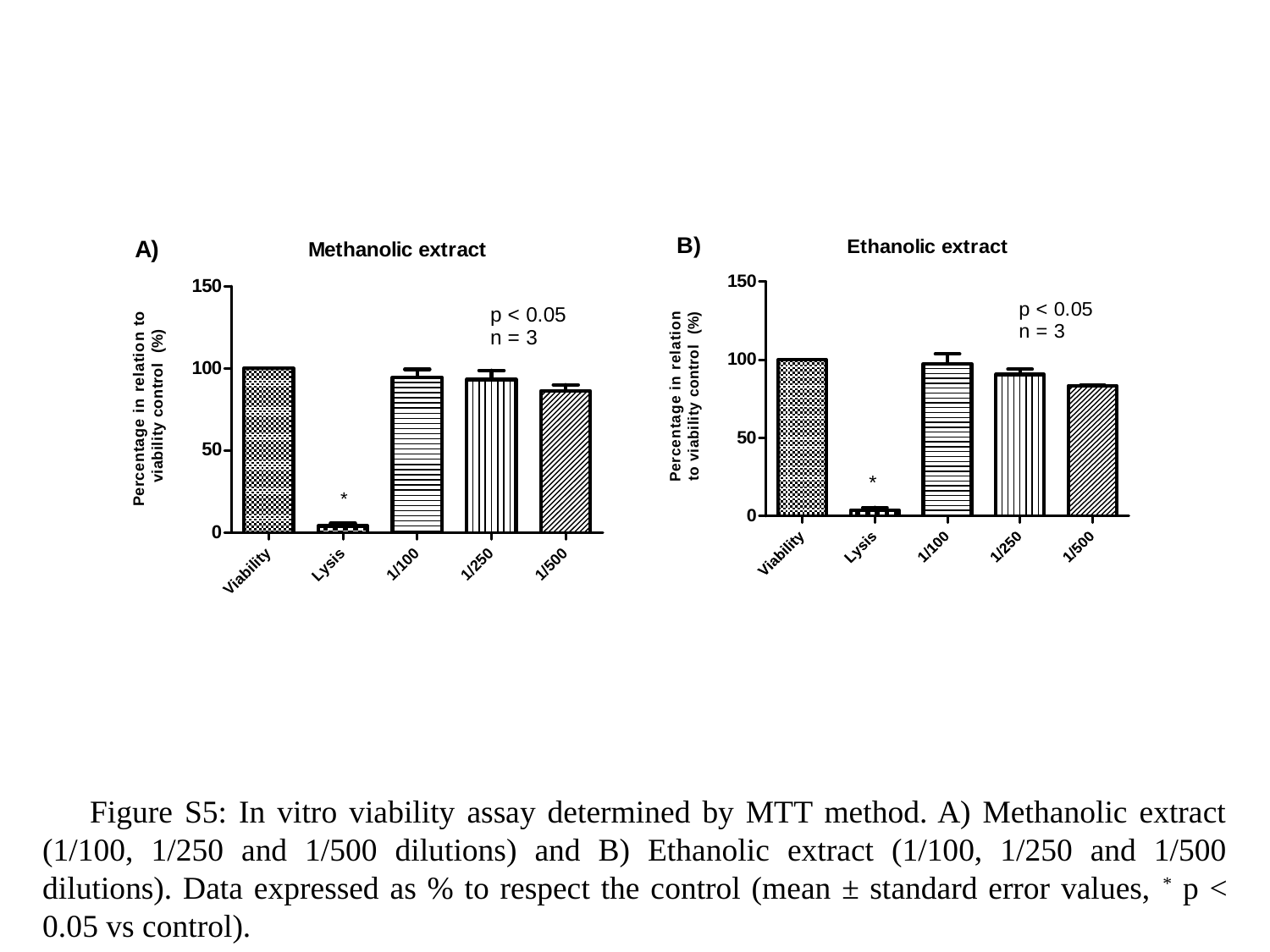

Figure S5: In vitro viability assay determined by MTT method. A) Methanolic extract (1/100, 1/250 and 1/500 dilutions) and B) Ethanolic extract (1/100, 1/250 and 1/500 dilutions). Data expressed as % to respect the control (mean ± standard error values, * p < 0.05 vs control).

## Slide 10
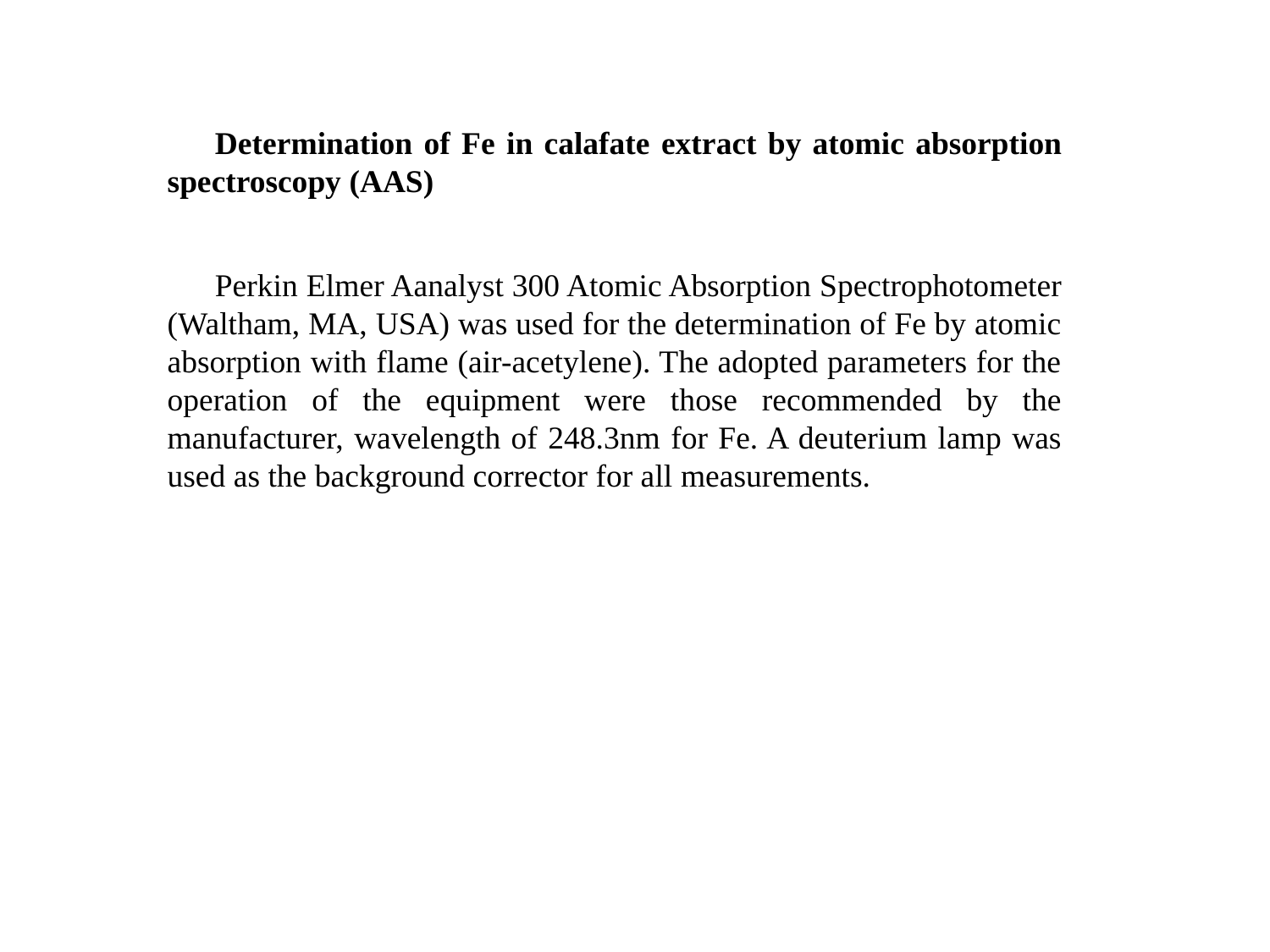

Determination of Fe in calafate extract by atomic absorption spectroscopy (AAS)
Perkin Elmer Aanalyst 300 Atomic Absorption Spectrophotometer (Waltham, MA, USA) was used for the determination of Fe by atomic absorption with flame (air-acetylene). The adopted parameters for the operation of the equipment were those recommended by the manufacturer, wavelength of 248.3nm for Fe. A deuterium lamp was used as the background corrector for all measurements.
